# Finding space for rewilding: Nature futures scenarios reveal ecological opportunities based on plural values of nature from participatory processes

**DOI:** 10.1371/journal.pone.0351326

**Published:** 2026-07-08

**Authors:** Laura C. Quintero-Uribe, Henrique M. Pereira, Rowan Dunn-Capper, Jenny Schmidt, Néstor Fernández

**Affiliations:** 1 Research Group of Biodiversity Conservation, German Centre for Integrative Biodiversity Research (iDiv), Halle-Jena-Leipzig, Germany; 2 Department of Zoology, Institute of Biology, Martin Luther Universität Halle-Wittenberg, Halle, Germany; 3 InBIO (Research Network in Biodiversity and Evolutionary Biology), University do Porto, Vairão, Portugal; 4 CoKnow Consulting – Coproducing Knowledge for Sustainability, Jesewitz, Germany; Mykolas Romeris University: Mykolo Romerio Universitetas, LITHUANIA

## Abstract

Ecological restoration is increasingly emphasised by ambitious biodiversity policies as essential for reversing ecosystem degradation in human-dominated landscapes. Among restoration approaches, rewilding fosters self-sustaining ecological processes and reduces the need for direct human intervention. However, effective implementation requires careful evaluation of co-benefits and trade-offs with local communities’ interests. This underscores the urgent need for participatory processes that align restoration efforts with local contexts. This study operationalised the Nature Futures Framework, a participatory, value-based scenario-planning methodology, to co-develop rewilding scenarios with local rewilding experts. To assess trade-offs and co-benefits, relevant stakeholders were systematically identified using Net-Map analysis. Semi-structured interviews and participatory mapping were used to assess stakeholder perceptions and the feasibility of translating scenario narratives into rewilding actions. The three scenarios articulated distinct priorities. The Nature for Nature scenario prioritised restoring landscape connectivity and promoting natural succession toward old forests. It also restored natural disturbance regimes, reflooded peatlands, restored historical river courses, and expanded protected areas to 65.8% of the region. The Nature for Society scenario emphasised reduced land use intensity, sustainable forestry, organic agriculture, and participatory flood management, covering 14.4% of the area. The Nature as Culture scenario highlighted community stewardship, biodiversity-friendly agriculture such as paludiculture, and the reintroduction of culturally significant species, encompassing 8.6% of the area. All scenarios converged on restoring water dynamics and improving connectivity. Spatial mapping identified both unique and overlapping priority areas. Overlaps near natural parks, grasslands, and settlements created high-value mosaics that support biodiversity, water regulation, and cultural services. These areas offer opportunities for risk management, sustainable agriculture, and the restoration of ecosystem services. Incorporating stakeholder perspectives enabled the identification of synergies and conflicts, guiding the development of context-specific strategies. Integrating scenario planning with participatory mapping strengthens adaptive restoration planning and provides a replicable model. This approach aligns rewilding actions with ecological, social, and cultural objectives, delivering tangible benefits for biodiversity, local communities, and policy implementation.

## Introduction

There is a growing recognition of the urgent need to halt biodiversity loss and reverse ecosystem degradation, leading to increased support for nature restoration as a key component of conservation strategies. While global agreements, such as the Kunming-Montreal Global Biodiversity Framework (GBF) and the European Nature Restoration Law (ENRL) (Regulation 2024/1991), demonstrate strong policy commitments, translating these commitments into measurable, effective results remains challenging. This is primarily due to the lack of consistent approaches for restoration planning and for understanding interactions between ecological and social factors [1]. Addressing this crisis requires actionable strategies that can convert high-level commitments into tangible, effective outcomes.

Rewilding is an emerging paradigm in restoration science that offers an innovative approach to address these challenges. It provides flexible, open-ended methods that can adapt to the dynamic and complex landscapes of today, fostering resilience at a ecosystem scale [2–4]. Unlike traditional restoration approaches prescribe fixed ecological endpoints and require active management for specific target species or habitats [5,6], rewilding does not establish a predefined target regarding the desired structure of the landscape or the composition of biodiversity [7].

Perino et al. 2019 [8] present a framework for rewilding that consists of three main components: the restoration of trophic complexity, the facilitation of natural disturbances, and the restoration of species dispersal processes. The restoration of the first component involves reintroducing species, either actively or passively, to restore important ecological interactions that have been lost. The facilitation of natural disturbances refers to the concept of allowing ecological events, such as fires or floods, to occur without human intervention. Lastly, the restoration of dispersal processes refers to the ability of species to move freely within the landscapes, promoting natural movement and population dynamics.

Effective rewilding initiatives require understanding the diverse relationships between nature and society [9,10]. Recognising that there is no one-size-fits-all approach, it is essential to consider how different societies perceive and engage with rewilding practices. Incorporating stakeholders directly into planning and assessment is therefore essential, not only for tailoring strategies to local contexts but also for supporting effective decision-making. Participatory scenario development provides a practical methodological approach for integrating diverse perspectives, fostering dialogue, and addressing uncertainty. This approach is increasingly recognised in scientific literature and is identified in the Intergovernmental Science-Policy Platform on Biodiversity and Ecosystem Services (IPBES) Kunming-Montreal GBF as a key process for conservation planning and monitoring [11,12].

Although participatory scenario planning is increasingly utilised in biodiversity conservation, the IPBES methodological assessment has identified persistent limitations in current approaches. Many widely adopted frameworks do not explicitly focus on nature, fail to articulate positive future scenarios, and inadequately address inclusivity or cross-scale integration [13]. These limitations hinder the integration of diverse values and stakeholder perspectives in ecological restoration planning and scenario development. To directly address these limitations, the IPBES expert panel introduced the Nature Futures Framework (NFF), a flexible, interdisciplinary, and systems-based approach. The NFF provides a structured conceptual and methodological foundation for scenario development, enabling the integration of multiple values through participatory processes while considering complex interconnections among biodiversity, ecosystems, and human well-being. By supporting the envisioning, assessment, and navigation of alternative pathways toward sustainability, the NFF advances more effective and equitable decision-making in restoration planning [14].

The NFF operationalises scenario development by integrating three foundational value perspectives: intrinsic, instrumental, and relational.The NFF distinguishes ‘Nature for Nature’ (intrinsic), which recognises the inherent worth of biodiversity; ‘Nature for Society’ (instrumental), which emphasises the benefits and services ecosystems provide; and ‘Nature as Culture’ (relational), which highlights the reciprocal relationships between people and nature shaped by cultural, spiritual, and social connections [15,16]. Grounding the framework in these perspectives facilitates the exploration of diverse human–nature relationships and supports the development of context-relevant scenarios [17,18]. By integrating participatory and co-design processes, the NFF offers a holistic tool for rewilding scenario planning that embraces multiple value perspectives and directly supports the development of effective and equitable restoration strategies [8,19–22].

We selected the Oder Delta as a case study site due to its status as one of the 30 officially designated biodiversity hotspots in Germany. This region offers exceptional rewilding opportunities, given the ongoing natural recolonisation of large mammals such as lynx, bison, and moose from neighbouring Poland, alongside economic opportunities and local population support for compatible land uses, including tourism and shift towards sustainable agriculture practices [23]. However, significant degradation of flood and water systems has increased vulnerability to climate impacts such as droughts, creating tensions between conservation aspirations and local livelihoods [24]. This convergence of high biodiversity value, diverse stakeholder interests, and competing land-use pressures creates a complex socio-ecological landscape where multiple visions of nature futures coexist, making the Oder Delta ideal case study for exploring how participatory approaches can navigate the co-benefits and trade-offs inherent in rewilding interventions.

Building on this context, the primary objective of this study is to assess how participatory scenario development that envisions diverse nature futures can improve understanding of the co-benefits and trade-offs associated with rewilding interventions. This study specifically examines how such processes can clarify uncertainties and inform adaptive management strategies to address emerging challenges and opportunities. Guided by the NFF, the research evaluates how multiple value perspectives shape restoration outcomes in complex socio-ecological settings. This approach addresses a critical knowledge gap in ecological restoration, where conventional planning often overlooks local ecological knowledge, resulting in implementation barriers and the emergence of new conflicts. This study represents the first practical application of the NFF at a local scale for rewilding and restoration in Europe. The methodological framework presented offers a transferable approach for navigating the socio-ecological complexities of European rewilding landscapes, supporting more equitable and ecologically effective restoration strategies.

## Methods

### Study area

**Fig 1:**
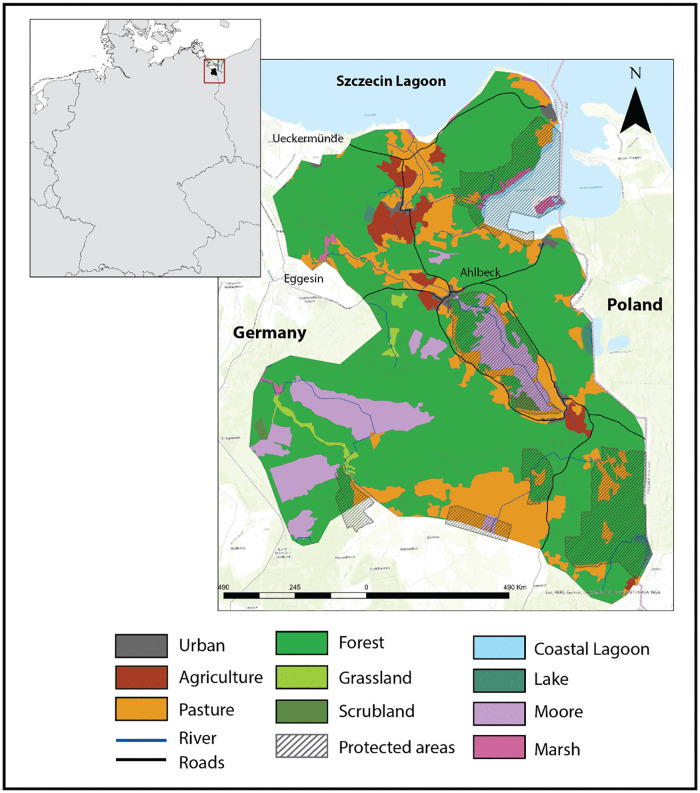
Map of Uckermünde Heath region in the Oder Delta region and the main land uses. © basemap.de / BKG (2024) CC BY 4.0 [25]; Administrative boundaries:geoBoundaries CC BY 4.0 [26]; Protected areas: European Environment Agency (EEA) CC BY 4.0 [27]; Land cover: Contains modified Copernicus Service information (2024) [28].

The Oder Delta region, situated along the Polish-German border at the confluence of the Oder River and the Baltic Sea, covers approximately 450,000 hectares and includes the Stettin Lagoon and a diverse range of ecosystems (Fig 1). The region is recognised for its high biodiversity, encompassing peatlands, marshes, wet meadows, and forests, and supporting species such as the European bison, Atlantic sturgeon, and white-tailed eagle. Numerous Natura 2000 sites and bird protection areas underscore its importance for migratory birds. Although the soils are generally poor, agriculture and forestry remain the dominant land uses; wheat cultivation and livestock production, primarily cattle and pigs, are central, with permanent grassland comprising 20% of the area. The agricultural landscape is currently undergoing a major transformation, characterised by a shift toward mixed forests, an increase in organic agriculture, and greater reliance on rapidly changing regional water dynamics [29–31].

The Oder Delta currently serves as a centre for ecotourism activities, including birdwatching, hiking, and swimming, while maintaining its role as a biodiversity hotspot and breeding ground for numerous species. Previous hydrological modifications and land use changes have resulted in habitat fragmentation, diminished ecosystem services, and reduced carbon storage. Historic water-diversion infrastructure, combined with the effects of climate change, has lowered the water table and heightened drought risk, threatening both agriculture and ecosystems. As a result, restoration and recovery of the water regime are considered urgent priorities for achieving climate neutrality by 2040 [24].

This study focuses on the Ueckermünde Heide (7,670 hectares), one of the largest forest complexes in the region. The area was selected for its diverse habitats, which include pine and beech forests, alder swamps, bogs, and dunes, making it central to regional rewilding initiatives. Current priorities include expanding beech forests and converting non-native conifer plantations to native mixed stands. Additional measures involve restoring moors, raising drainage thresholds on key streams, and transitioning to free-flowing water systems. These initiatives seek to balance ecological restoration with local needs, such as allowing firewood collection and ecologically oriented hunting for wildlife management, thereby positioning the Ueckermünde Heide as a model for integrating rewilding, climate neutrality, and resilience [32].

### Terminology and definitions

To ensure methodological clarity, this section defines the principal terms employed throughout the manuscript and the methodological framework for participatory scenario development. “Scenarios” are broad, plausible representations of future socio-ecological systems. They are constructed to explore alternative pathways. “Scenario storylines” are the qualitative narratives or plots that outline how key drivers and uncertainties may shape these futures. Embedded within each storyline, “scenario narratives” provide detailed, context-specific visions. These narratives capture the diversity of human-nature relationships and values. “Scenario narrative elements” are the specific qualitative details and locally relevant themes identified through stakeholder engagement [14,33]. These are incorporated into the narrative construction. These definitions structure the design of participatory scenarios and inform the application of “rewilding.” Rewilding is an approach to restoration that emphasises ecosystem complexity at multiple scales. It seeks to restore self-sustaining ecological systems by recovering lost species interactions and ecosystem functions. Rewilding focuses on three core components: trophic complexity, natural disturbances, and dispersal. Rewilding actions are practical, site-specific interventions such as species reintroductions or barrier removals. These actions directly address and operationalise these components within each scenario. They serve as tangible elements that restore ecological integrity and reduce human impacts [2,8,22].

### Participatory scenario development process

**Fig 2:**
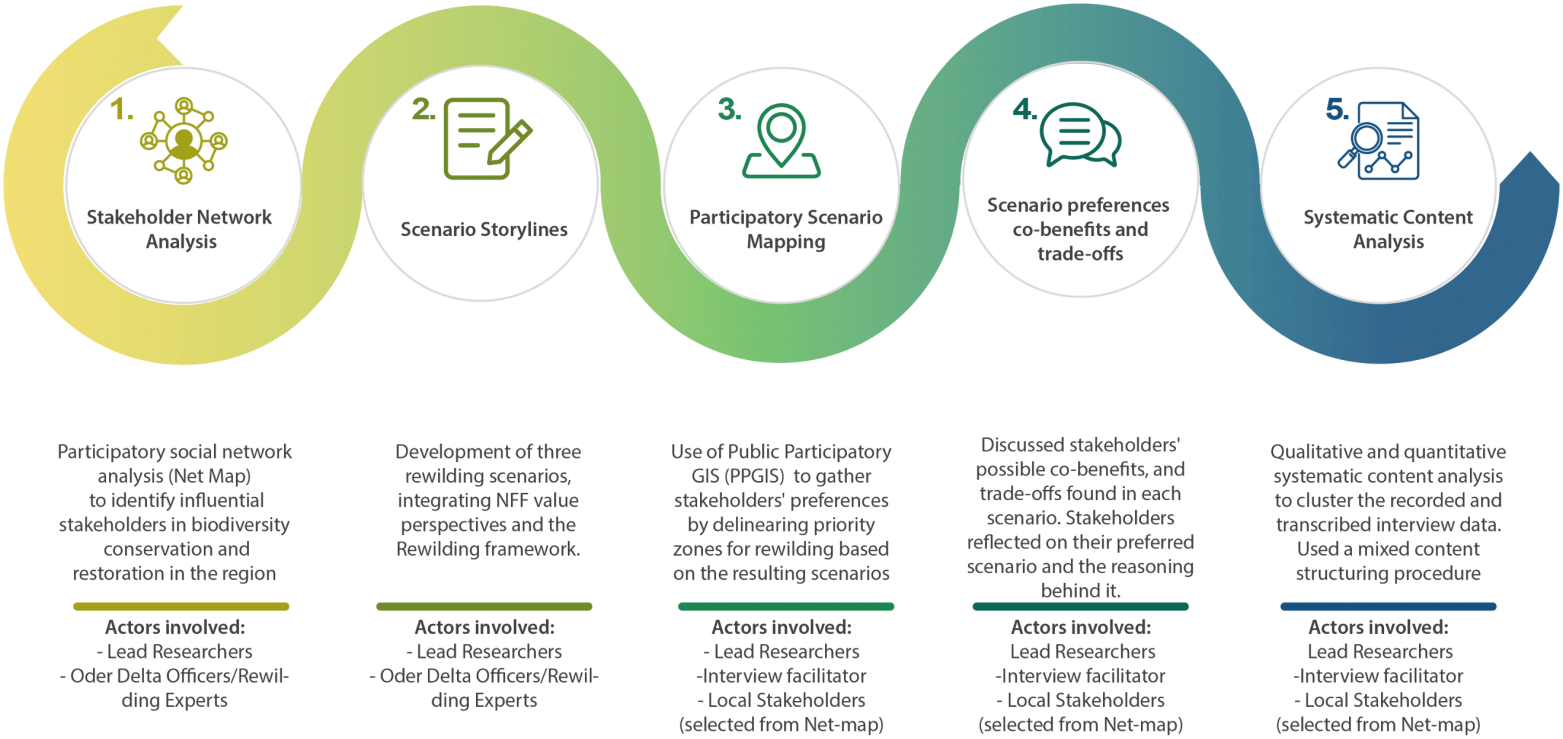
Flowchart illustrating the detailed steps undertaken during scenario development and assessment, including information on the actors involved at each stage of the process. Note: “Network” icon by Ifanicon, “Storylines” icon by Ilham Fitrotul, “Map” icon by Ibad Syukron, “Preferences” icon by David Khai, and “Contingent analysis” icon by Miftahul Huda from thenounproject.com (CC BY 3.0).

In the preliminary phase, officials from the Rewilding Oder Delta e.V. (ROD) organisation and experts in rewilding were involved in co-developing the study's objectives and guiding the selection of key regional stakeholders, ensuring alignment with regional challenges, regulations, opportunities, and priorities. We carried out the scenario development process in four phases: 1) participant identification and sampling; 2) development of scenario storylines; the semi-structured interviews which consisted of two steps 3) participatory scenario mapping and 4) assessment of scenario preferences, co-benefits and tradeoffs; and 5) data analysis through systematic content analysis (Fig 2).

### Participant identification and sampling

To identify the group of stakeholders for our interviews and scenario assessment exercises, we employed the Net-Map tool to discern influential actors in the region. In this context, we decided to interview rewilding experts and practitioners from the Rewilding Oder Delta Team e.V., given their extensive knowledge of the regional conservation landscape. This approach enabled us to capture a diverse array of stakeholders engaged in local decision-making processes, enhancing our understanding of the intricacies of socio-ecological dynamics within the community.

The Net-Map tool itself integrates social network analysis with a focus on power relations, structured into a five-step process (Schiffer and Hauck, 2010). Initially, we framed our guiding research question: “Who are the most influential actors in biodiversity conservation and sustainable landscape management in the region?” This inquiry helped pinpoint stakeholders who not only influence decision-making but also possess insights into local dynamics, serving as intermediaries between regional governance and ground-level realities.

Subsequently, we mapped out relationships among identified actors by using color-coded cards on large maps to represent various stakeholder groups, such as governmental agencies, NGOs, civil society organisations, and private sector entities. Relationships were illustrated with arrows that indicated information exchange, advice provision, and collaborative efforts, with variations in arrow thickness reflecting the strength and importance of each connection. The resulting network maps allowed us to identify particularly influential or strategically positioned stakeholders based on their centrality and relational strengths, which informed our selection for in-depth interviews aimed at evaluating rewilding scenarios through participatory processes. This structured approach not only facilitated a thorough stakeholder analysis but also provided valuable insights into the dynamics shaping biodiversity conservation and sustainable landscape management in the region.

### Development of scenario storylines

The research team, in collaboration with the Rewilding Oder Delta e.V., developed the scenario narratives tailored to align with local priorities. We applied the methodology outlined by [22] which maps rewilding elements and Nature contributions to people within the NFF to construct the storylines. The NFF served as a guiding framework to structure the scenarios around the principle of pluralistic values, effectively capturing three distinct perspectives: Nature for Nature (intrinsic values), Nature for Society (instrumental values), and Nature as Culture (relational values) [14]

For each scenario, we mapped the core components of rewilding, including trophic complexity, landscape connectivity, and stochastic disturbances, and linked them to the corresponding value perspective. This mapping process required us to adapt each rewilding practice according to the underlying values. For example, in the “Nature for Nature” scenario, we prioritised actions that promote biodiversity conservation and emphasise intrinsic ecological values. In the “Nature for Society” scenario, we tailored actions, such as restoring water dynamics, to highlight their regulatory and provisioning benefits, thereby supporting future water security. Meanwhile, the “Nature as Culture” scenario focused on practices that enhance community engagement and landscape stewardship, reinforcing relational and cultural values while simultaneously conserving biodiversity (Table 1).

**Table 1:** Key elements from the overall scenario storylines.

		Scenario		
		Nature for Nature (NfN)	Nature for Society (NfS)	Nature as Culture – One with Nature (NaC)
**Main narrative**		Nature comeback – All rewilding actions are centred on restoring lost species interactions and ecosystem functions.	Maximising nature contributions to people- rewilding to enhance nature's benefits to people through sustainable management.	Living in harmony with nature- Community-based rewilding for local identity and stewardship
**Action**	**Component**	**Nature for Nature (NfN)**	**Nature for Society (NfS)**	**Nature as Culture – One with Nature (NaC)**
**Rewilding framework (Perino et al.,)**	**Trophic Complexity**	Strict actions for habitat protection and keystone species rehabilitation (e.g., nesting programmes)	Reintroduction of species with a focus on restoring ecosystem services (e.g., pest regulation through predation and pollination).	Restoring trophic complexity through return of emblematic species (e.g., bison, elk and grey seal)
	**Connectivity**	Enhancing connectivity with green and blue corridors to link protected and high biodiversity value areas	Promoting connectivity through adaptive management practices.	Promoting connectivity for diverse and heterogeneous biodiversity-friendly landscapes
	**Natural Disturbances**	Rewilding efforts focus on rewetting dried peatlands to restore natural stochastic dynamics and biodiversity conservation.	Restoring natural disturbances to improve water and soil quality in peatlands	Community-based rewetting management for natural disturbances and biodiversity-friendly practices
**Nature contributions to people**	**Regulatory services**	Regulation of freshwater quality through the restoration of riverbeds and floodplains	Regulating freshwater quantity and flooding regimes through adaptive management	Climate regulation through the restoration of peat soils for CO2 absorption
	**Material Services**	Reducing intensity of farming and wood extraction. Improvement of water resources.	Intensive farming through precision agriculture; sustainable wood extraction	Organic farming and paludiculture g for renewable energy and construction materials
	**Non-material services**	Improvement of well-being through the creation of new spaces for enjoyment of natural landscapes	Improving the local economy through sustainable management and technology in agriculture	Landscape stewardship, community-based rewetting, and youth engagement for biodiversity-friendly farming

^a^Note: This table summarises key rewilding elements and Nature's Contributions to People described in the scenarios. See S1 Table for details.

We repeated this process for each rewilding component and each Nature contribution to people,regulatory, material, and non-material,resulting in approaches that aligned with each value perspective. The scenario development produced three distinct storylines, one for each NFF perspective, illustrating how the landscape in the study area would evolve over 30 years under each scenario (Table 1). We chose a 30-year time frame for our study, setting the horizon at 2050 to align with critical policy timelines, including the European Nature Restoration Regulation and the goals of the Kunming-Montreal Biodiversity Framework, both of which aim to achieve significant environmental targets by 2050. We subsequently shared these storylines with stakeholders to incorporate local knowledge, foster discussions, and evaluate potential benefits and trade-offs associated with various rewilding strategies. Additional details regarding the specific scenario elements and the resulting storylines are available in the supplementary material S1 Table.

### Semi-structured interviews

We conducted stakeholder interviews from September 2021 to March 2022 through online video calls, required by COVID-19 guidelines at the time. The scenario narratives and storylines were completed prior to the interviews, which allowed us to present them effectively to the stakeholders selected from the net map analysis. We carried out one-hour semi-structured online interviews via videocall, structured in four phases: 1) rapport-building, 2) introduction to the scenario storylines, 3) participatory mapping, and 4) discussion of scenario preferences, co-benefits, and trade-offs (S2 Table).

1) The initial step involved rapport-building, during which the interviewer introduced the purpose of the interview, provided an overview of the procedure, and obtained informed consent from the participant. This stage aimed to establish trust and ensure respondent comfort. Given the nature of this study, and in compliance with university guidelines at the time, the lead supervisor responsible for the project granted ethics approval for this study. The study adhered to the German Research Centre for Integrative Biodiversity Research's code of conduct and General Data Protection Regulations (GDPR). All interviewed participants provided informed consent via a written form, in which they signed their approval regarding the data management and storage procedures, ensuring confidentiality and the protection of data. A consultancy firm supported participatory processes, ensuring that all necessary steps were taken to avoid putting stakeholders at risk or in vulnerable positions. To protect participants discussing sensitive topics like rewilding, we anonymised the interviews and stored the raw data securely in password-protected folders accessible only to the principal investigators.
2) Following this, we presented the narrative of the scenarios to the participants as short stories that captured the main land use drivers of change. We guided the participants to envision changes in the local landscape over the next 30 years. Importantly, we instructed the participants to set aside current land ownership and political contexts, allowing them to focus more fully on potential transformations within the landscape.
3) Participatory Mapping: We utilised participatory mapping to gather stakeholder preferences for future land use, allowing participants to visualise landscape changes [34]. We asked stakeholders to map out how land use types will change over 30 years based on four action categories: changes to protected areas, reflooding zones, the creation of green and blue corridors for connectivity, and promoting biodiversity-friendly agricultural practices. During this process, we encouraged participants to identify areas of interest, highlight potential conflicts, and suggest preferred rewilding actions on the provided maps. The template utilized during the participatory scenario mapping process is provided in S1 Fig.
4) Assesment of stakeholder Preferences: Finally, we asked the stakeholders to rank the scenarios from most preferred to least preferred. Following this, we asked participants to reflect on each scenario, asking them to identify what co-benefits and trade-offs they perceived for each scenario.

### Systematic content analysis

This study utilised two primary data inputs derived from interviews: the transcribed texts of the interviews and participatory mapping. In the first phase, we conducted a systematic content analysis that integrated both qualitative and quantitative methods to categorise and cluster the transcribed interview data [35]. We employed a mixed content structuring approach, combining deductive and inductive category formation to ensure a comprehensive synthesis of insights.

We initially defined deductive categories to cluster the information effectively. We grouped the scenario elements into two main clusters: rewilding components, which include trophic complexity, landscape connectivity, and stochastic disturbances, and NCPs, which encompass regulatory, material, and non-material aspects. Our objective was to identify potential rewilding actions that we could implement in the study region.

To refine the analysis, we synthesised the information into subcategories that outlined specific rewilding practices corresponding to each category definition. This synthesis involved a meticulous line-by-line examination of the transcribed material. Upon identifying relevant content that fit the predefined category definitions, we constructed subcategories labelled with terms or brief phrases that encapsulated the associated rewilding actions.

After completing the interview analysis and confirming that no new categories emerged, we reanalysed the transcripts to quantify how often terms occurred throughout the interviews. This quantitative aspect was crucial for gathering qualitative insights, acting as a proxy for stakeholders’ preferences regarding the potential implementation of rewilding actions in the study region. The database encompassing the coding framework and classification system employed for the systematic extraction of qualitative data from the interviews is accessible in the following data repository https://doi.org/10.5281/zenodo.18637351.

In the second phase, based on the outcomes of the participatory mapping process, stakeholders were asked guiding questions by the facilitator to map the spatial allocation of the scenarios within the study area. Participants identified and marked various actions for each scenario on a map, specifically indicating: 1) changes to protected areas, 2) areas designated for reflooding, 3) green and blue corridors to enhance connectivity, and 4) pathways toward adopting more extensive or biodiversity-friendly agricultural practices. Following this mapping exercise, a total of nine distinct maps emerged, each representing the actions identified by stakeholders for their respective scenarios. We used ArcGIS Pro 3.5.2 software to digitise these maps, creating three comprehensive maps—one for each scenario—that reflect the cumulative input from stakeholders.

To analyse the spatial data, we developed heat maps for each scenario, illustrating the frequency with which stakeholders marked particular areas. High-preference areas showed significant overlap in markings from multiple stakeholders, whereas low-preference areas reflected input from only one or two participants. Subsequently, the analysis involved overlaying the heat maps generated from each scenario, which facilitated the creation of a comprehensive map illustrating the frequency of intersections among the different scenarios. This resulting map enabled a categorisation based on the extent of overlap: areas where only one scenario was identified, those where two scenarios intersected, and regions where all three scenarios converged. The systematic approach revealed key insights into the interactions between scenarios, identifying regions with unique characteristics and highlighting areas of stakeholder agreement and disagreement regarding land-use actions. This analysis provided a clear visual representation of potential land-use changes in the Oder Delta, reflecting the values and preferences of stakeholders.

## Results

The participatory process outcomes are organised into two main sections. The first section analyses the rewilding actions identified in each scenario through a systematic content analysis. The second section presents the results of the analysis of the outcomes from the participatory mapping.

### Net map outcomes

The Net-Map analysis provided important insights into how various stakeholders interact regarding biodiversity conservation and sustainable landscape management in the region. In the initial phase, rewilding experts and practitioners identified 17 potential actors they perceived as key to biodiversity conservation efforts. These actors were categorised into the following groups: six from civil society associations related to forestry and agriculture, five from NGOs focused on biodiversity conservation, four from public administration institutions, and two affiliated with scientific research and academia. The analysis illustrated relationships among these stakeholders using marked arrows to indicate information exchange, advice provision, and collaboration. Notably, the Deutsche Bundesstiftung Umwelt (DBU) and the Natural Park Stettiner Haff emerged as pivotal actors, alongside the NGOs of Rewilding Oder Delta and the Naturschutzbund Deutschland e.V. (NABU), all of which demonstrated substantial relational strengths (S2 Fig).

Based on the identified relationships, we recognised seven influential actors, indicated by darker arrows in the analysis, who play critical roles in decision-making and actions related to biodiversity conservation and restoration. These key actors include organisations such as DBU, NABU, and the Natural Park Stettiner Haff. Other key actors include actors from the tourism sector, government officials linked to Department of Nature and Landscape Protection and civil associations focused on forestry and organic agriculture. Utilising the Net-Map analysis to assess the actors’ relationships and influence, we selected a pool of stakeholders for in-depth interviews (S3 Table). Despite COVID-19 restrictions, we successfully interviewed nine stakeholders and stopped the interviews when data collection saturation was reached. The final nine participants we selected come from diverse sectors, including governmental organisations, academia, forestry, organic agriculture, NGOs, and natural park administration, representing a well-rounded and varied group (Table 2).

**Table 2:** Stakeholder selection and relationship mapping from Net-Map analysis. The table lists nine interviewed stakeholders, their mapped information flow relationships, and actor relevance, measured by influence on information sharing and decision-making (darker arrows indicate stronger influence).

Mapped relationships (arrows)	Relevance (Darker arrows)	Stakeholder Group	Working area
12;3 *	8;1	Conservation NGO, Civil society association	Biodiversity conservation, Eco-tourism
9	5	Conservation NGO	forest conservation
8	7	Public Administration	Tourism -biodiversity conservation-land management
8	4	Conservation NGO	Biodiversity Conservation
7	2	Public Administration	Biodiversity Conservation
7	2	Public Administration	Biodiversity Conservation
4;2 *	1;0	Conservation NGO; Academia	Water Management
3	3	Civil society association	Sustainable Agriculture sector
3; 2 *	3; 0	Civil Society association	Forestry – Hunter Sector

Note: Some of the actors represented more than one group.

* two numbers indicate two different actors, each with their own values for mapped relationships and relevance.

### Overview of rewilding actions identified for each scenario

**Fig 3:**
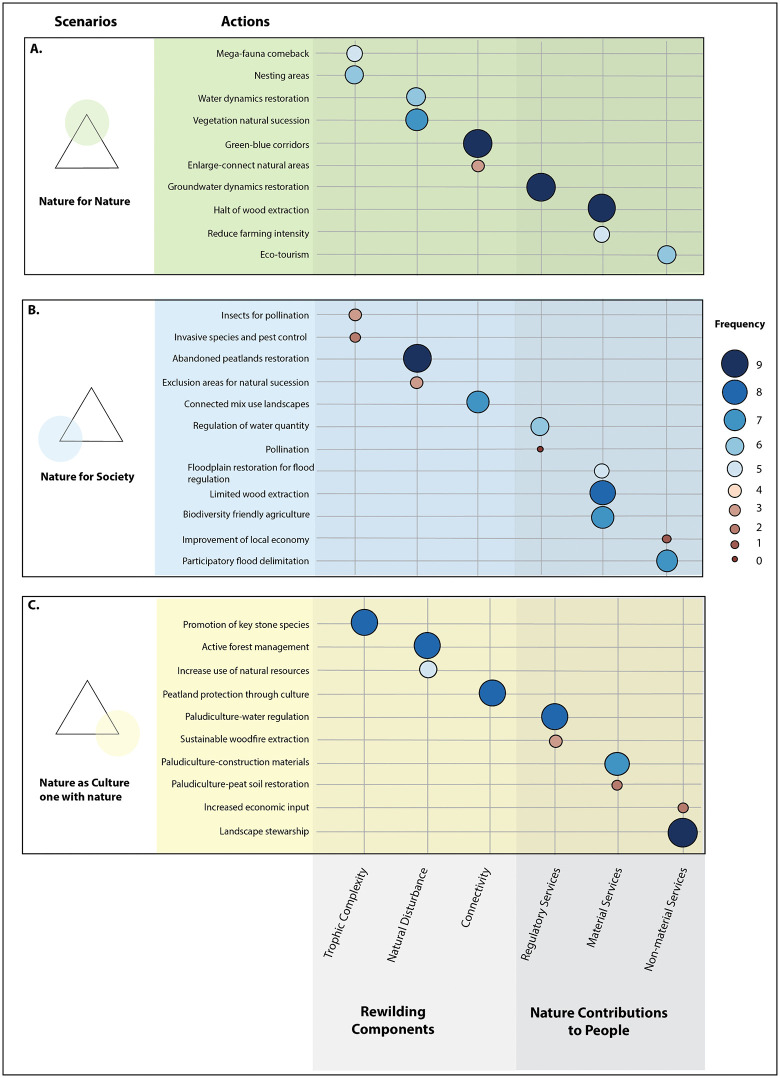
Frequency of main actions mentioned by the stakeholders for the different NFF scenarios. Circle size and colour indicate the frequency with which specific actions were mentioned across scenarios: smaller red circles correspond to less frequently mentioned actions, while larger, darker blue circles represent actions cited more often.

Under a Nature for Nature scenario (NfN), the key rewilding components arising from the resulting narratives is the prioritisation connectivity and natural disturbance restoration to increase wildness; the focus is on protected and other ecosystems under low human resource use. Stakeholders identified the creation of blue and green corridors as a primary target of rewilding, with two key benefits: facilitating the free movement of species and restoring the region's long-lost water dynamics (Fig 3A). Furthermore, stakeholders concurred in prioritising actions aimed at achieving self-regulated ecosystem processes in both terrestrial and freshwater realms. These included halting wood extraction in some forested areas, recovery of natural groundwater dynamics, and reflooding dried peatlands. A smaller group of stakeholders mentioned actions to restore trophic complexity in the region, notably breeding and feeding habitat restoration programs for the lesser spotted eagle (Fig 3A). Stakeholders indicated some indirect benefits of rewilding to people with emphasis on the provision of better water quality and quantity, and carbon sequestration in peatlands (Fig 3A-NCPs section).

Under a Nature for Society scenario (NfS), the majority of stakeholders (more than 5) chose actions to reduce land use intensity by implementing technological advances. Similar to the previous scenario, rewilding was associated with restoring natural processes, e.g., allowing for natural succession in grasslands and rewetting drained peatlands currently not used for agriculture. However, the main emphasis was on the sustainability of material contributions to people through regulating wood extraction and biodiversity-friendly agriculture, such as adopting precision agricultural production to reduce pollutants (Fig 3B). Similarly, non-material nature contributions, such as participatory flood delimitation, were actions often mentioned by the interviewed stakeholders. Other important but less frequently mentioned actions include regulatory services such as flood regulation and water dynamics regulation. In this scenario, protected areas primarily aim to restore grasslands and forests by actively harnessing natural succession in non-productive lands.

In the “Nature as Culture" (NaC) scenario, stakeholders emphasised the need to implement co-production and community-based actions. Rewilding initiatives aimed to promote landscape stewardship, support local economies through biodiversity-friendly agriculture and reintroduce emblematic species to enhance local identities and cultural value. These initiatives often mention regenerative agricultural practices, such as sustainably harvesting reeds through paludiculture to provide raw construction materials (see Fig 3C). The stakeholders favoured the potential benefits of paludiculture for enhancing water quantity and sequestering carbon.

We found that stakeholders in all scenario narratives agreed on the need for actions that restore natural disturbance dynamics and bolster landscape connectivity through measures such as river and peatland reflooding (Fig 3).

### Mapping scenario storylines

**Fig 4:**
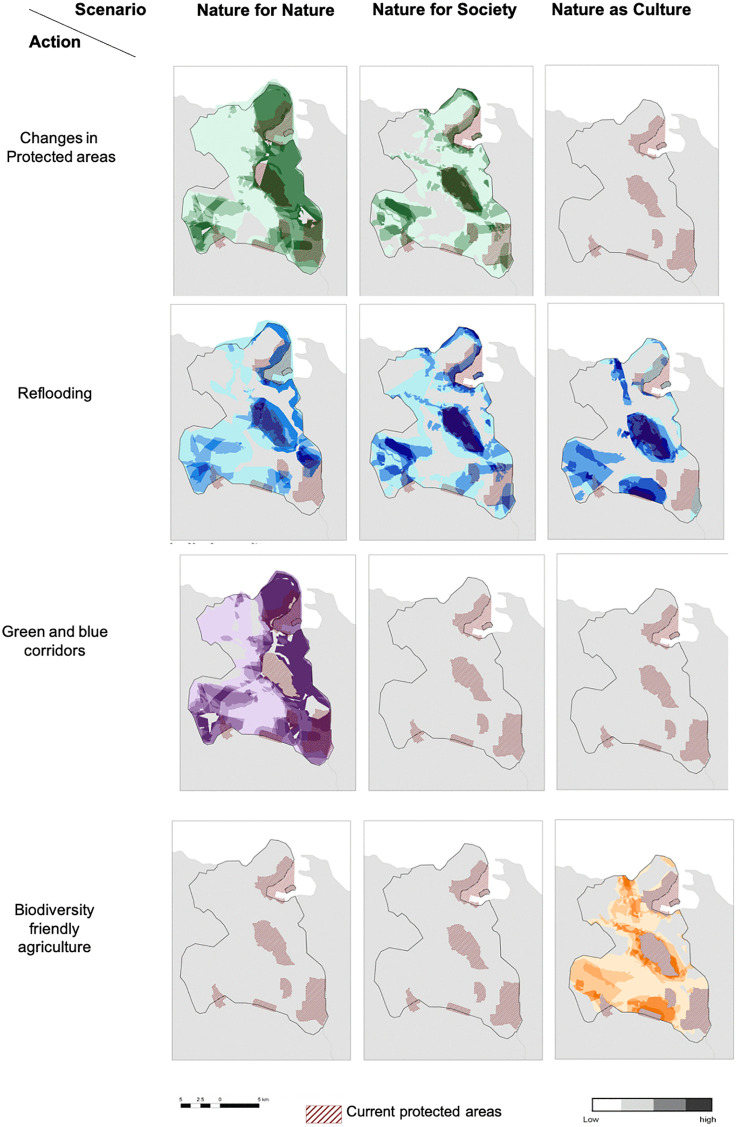
Heatmap of rewilding actions by scenario, as identified after overlapping the areas of interest identified by the stakeholders. Green areas for nature protection, blue areas for reflooding or restoration of water dynamics, orange areas for biodiversity-friendly agriculture, and purple areas for blue or green corridors facilitating biodiversity conservation. Darker colours represent areas identified by 8 or 9 stakeholders, while lighter colours are areas identified by 1 or 2 stakeholders. © basemap.de / BKG (2024) CC BY 4.0 [25]; Administrative boundaries:geoBoundaries CC BY 4.0 [26]; Protected areas: European Environment Agency (EEA) CC BY 4.0 [27].

The stakeholder mapping of scenario actions illustrated key priority areas for rewilding and land use management under the different NFF scenarios. We grouped areas into four major categories: expansion of protected areas and management changes, reflooding, creation of green and blue corridors, and biodiversity-friendly agriculture (Fig 4).

**Fig 5:**
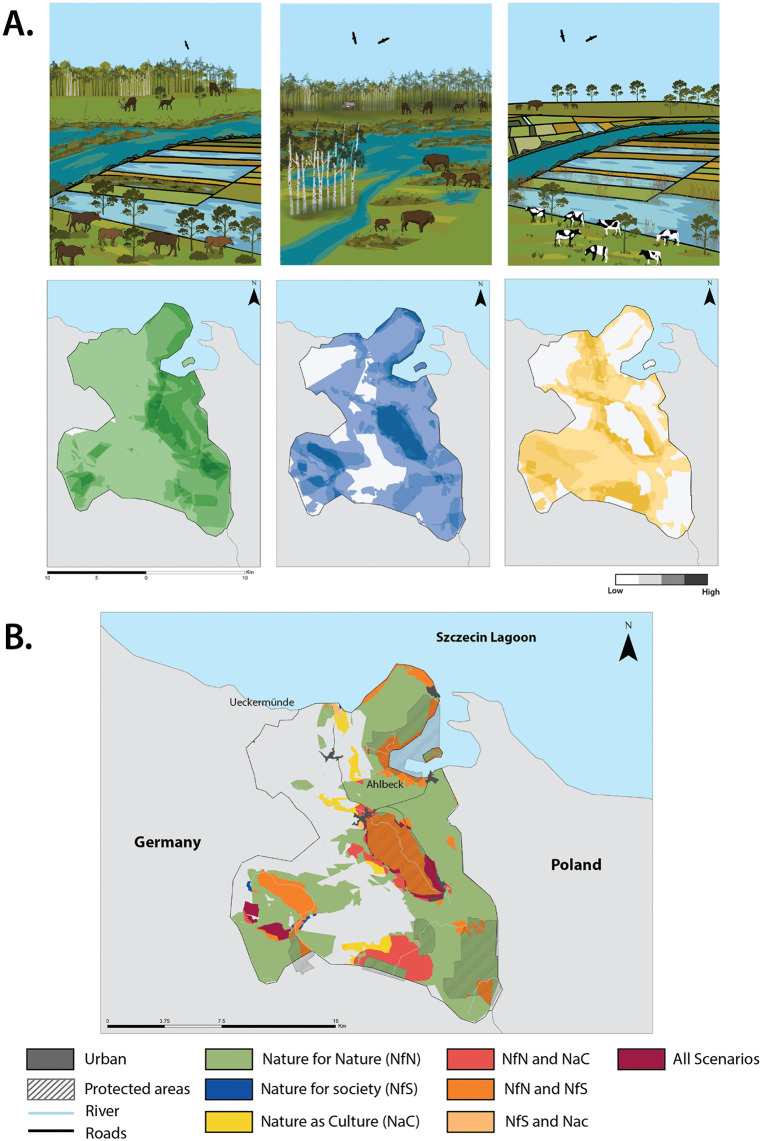
A. Consolidated map illustrating the spatial distribution of areas designated for biodiversity conservation, rewetting, blue-green corridors, and biodiversity-friendly agriculture across various scenario narratives. The colors represent each Nature Future Scenario: green for “Nature for Nature,” blue for “Nature for Society,” and yellow for “Nature as Culture.” Darker shades indicate areas identified by 8 or 9 stakeholders, while lighter shades represent areas identified by 1 or 2 stakeholders. **B.** Map illustrating the intersection of the three scenario maps found in section A. Areas are categorised based on their degree of spatial or physical overlap among the three scenarios, showing distinct zones associated with only one scenario, areas of intersection between two scenarios, and regions where all three scenarios converge. © basemap.de / BKG (2024) CC BY 4.0 [25]; Administrative boundaries:geoBoundaries CC BY 4.0 [26]; Protected areas: European Environment Agency (EEA) CC BY 4.0 [27].

For the NfN scenario, mapping resulted in large rewilding areas covering most of the study area (Fig 5A). Most of the actions took place along the border with Poland, an area characterised by extensive coniferous and broadleaf forests. Respondents identified key rewilding outcomes as higher forest and transboundary ecological connectivity, a larger old-growth forest cover, and the recovery of functional peatlands. Stakeholders prioritised the expansion of protected areas in roadless areas, highlighting that removing dikes and dams would restore connectivity in wetlands and create large, interconnected freshwater habitats during the mapping process. With these actions, an increase in connectivity across freshwater and terrestrial realms would benefit the passive restoration of the trophic complexity (Fig 4).

In the NfS and NaC scenarios, most actions aimed to de-intensify land management, although there were no major land-use changes. The NfS scenario extended protected areas, but these were smaller than in NfN and often allocated as buffer zones at the interface with farming and forestry areas (Fig 5B). Reflooding is prioritised in peatlands and grasslands along rivers and coastal areas to protect farmland and urban areas against floods. As a result, highly heterogeneous landscapes would dominate, shaped by diversified uses (Fig 4).

The NaC scenario transformed intensive agricultural areas into extensive forms, with widespread implementation of reflooding. Protected areas did not increase. Instead, reflooding was allocated in some farming areas to create new semi-natural habitats compatible with extraction activities (e.g., paludiculture). Areas surrounding croplands, grasslands, and rivers were designated for rewilding, creating a landscape mosaic (Fig 4).

When considering only the areas of interest mapped by five or more stakeholders (marked as darker green in Fig 5A), the total area allocated to NfN actions covered 65.8%, followed by NfS (= 14.4%) and NaC (= 8.6%). By converging the layers of the three scenarios on top of each other, we end up with a map that shows areas where two or three scenarios converge or areas where only one type of scenario is found (Fig 5B). For areas where only one type of scenario is found the narrative of NfN exclusively covered 47.2% of the landscape, followed by NaC with 2.36% and NfS with a total of 0.16% of area covered.

Regions where the NfN and NfS scenarios overlapped encompassed 14.06% of the study area (Fig 5B). Actions in these regions included strategies such as reflooding for disaster risk management and innovations to support biodiversity-friendly practices. The overlap between NfN and NaC, which only covered 5.6% of the total landscape, involved zones for transitioning to small-scale agricultural practices, which resulted in material provisions such as construction materials and food. The areas overlapping between NfS and NaC covered 2.13% of the total area (Fig 5B). These areas distributed along traditional grasslands, agricultural fields, and towns. The main rewilding actions associated with them included restoring natural water dynamics and enhancing agricultural yields.

The regions where all three scenarios spatially overlapped on the map covered only 1.91% of the total study area (Fig 5B). These areas were found adjacent to natural parks, extensive livestock grasslands, and urban settlements. As demarcated by the stakeholders, these areas hold a high nature value in terms of biodiversity conservation and providing services such as water quantity and cattle feed production. These smaller areas are distributed along the landscape, creating a mosaic between all three narratives.

## Discussion

The Nature Futures Framework (NFF) has been successfully implemented in large-scale studies [36,37], predominantly focusing on nature-centred narratives within Europe. However, translating these narratives into actionable rewilding efforts at the local level presents a significant challenge. Our research highlights the diverse and often conflicting values associated with rewilding in the Oder Delta, illustrating the complexity of applying broad, nature-centred narratives to specific landscape management situations.

Moreover, our findings indicate that considering multiple natural values greatly enhances rewilding outcomes. This approach reveals a spectrum of actions, ranging from passive to active rewilding interventions, which are essential for effective stewardship. By mapping the spatial overlap of various scenarios, we were able to identify priority regions for intervention, demonstrating the potential applicability of this method in other locations facing similar socio-ecological challenges. These results highlight the importance of integrating diverse values into rewilding strategies to promote adaptive, context-specific landscape management.

### Net Map a tool for stakeholders identification

The findings of this study highlight the value of Net-Map as a participatory tool for understanding the complex social dynamics present within socio-ecological systems. As emphasised by Schiffer and Hauck [38,39], the identification of stakeholders that act as ‘bridge builders’,intermediaries with cross-sectoral ties,proves pivotal for facilitating communication and trust between policy-makers and local stakeholders. The capacity of these actors to mediate interests and foster the integration of local perspectives into rewilding objectives underscores the necessity of inclusive governance for successful conservation. This is further illustrated by the documented collaborations among diverse actors, such as the implementation of grazing regimes to support bird habitat, cooperative efforts to manage water resources in response to drought [24], and the multi-actor ‘naure conservation and climate adaptation projects, which brought together forest associations, local stakeholders, and hunting associations in adaptive wildlife management for both deciduous forest and moorland restoration [40–42]. Our findings demonstrate that mapping actor relationships and information flows is essential for developing targeted, context-sensitive rewilding strategies, as it enables the design of adaptive and locally tailored interventions that are more likely to achieve long-term conservation success.

### Rewilding strategies proposed for each scenario

We developed three scenario narratives by focusing on key components of rewilding, creating a synergistic approach that improves upon previous participatory methods. Earlier scenarios often emphasised either the practical benefits of nature, such as ecosystem services, or the more protective intrinsic nature values, without integrating the two. This lack of integration is insufficient to achieve the transformative change needed to halt biodiversity loss, as supported by existing research [43]. During our discussions, we encouraged stakeholders to explore actions that balance intrinsic, instrumental, and relational values, fostering a dialogue around pluralistic perspectives that can coexist. This approach allowed us to view rewilding not merely as a nature-centric initiative [19,22], but as a multifaceted interplay of diverse values. This enabled us to identify transversal actions, such as rewilding initiatives that involve connecting landscapes and reflooding peatlands, which appeared in all three narratives. Additionally, we identified distinct management strategies tailored to each scenario, shaped by the value framework employed, reflecting the diverse needs and priorities of the local community.

Although each scenario reflected a distinct value perspective, all scenarios converged on similar rewilding actions, indicating that targeted interventions can generate multiple co-benefits across the landscape. For instance, the “Nature for Society” scenario prioritised peatland restoration, river reflooding, and reduced forestry to enhance water regulation and carbon sequestration [2,44]. The “Nature for Nature” scenario emphasised improving connectivity and restoring natural disturbance regimes, specifically through reconnecting fragmented landscapes and restoring old river courses and floodplains. While these measures primarily aim to enhance biodiversity and support self-sustaining ecosystems, they also provide indirect benefits to society by improving hydrological dynamics and regulating water availability and quality [45]. Nature as Culture” scenario, stakeholders favoured peatland rewetting to support extensive agriculture (i.e., paludiculture), while restoring natural hydrological processes and minimising human intervention [46]. These examples demonstrate that, regardless of underlying motivations, targeted rewilding can deliver both ecological and societal benefits.

The convergence of rewilding efforts across various scenarios can reveal important regional priorities for natural resource management and biodiversity conservation, while also highlighting the complexity in planning rewilding projects. Throughout our discussions with stakeholders, many highlighted the need to ensure long-term water resources to support agricultural productivity while safeguarding crucial ecosystems [47,48]. They emphasised the need to tailor diverse rewilding actions to address future challenges and foster resilience within the community. This situation reflects ongoing social and environmental pressures and the importance of adaptive land management strategies that can effectively respond to future changes.

A significant outcome of our project is that stakeholders predominantly associate keystone species reintroduction with cultural values. This observation contrasts with previous research, which has typically emphasised the intrinsic (NfN) or instrumental (NfS) value of trophic rewilding [49,50] and highlights the need for rewilding strategies that incorporate cultural aspects alongside ecological and economic considerations. Our findings reinforce existing literature that underscores the importance of involving local communities in rewilding efforts. Increased engagement with local communities has been identified as a crucial factor in fostering public acceptance of rewilding initiatives, particularly in relation to human-wildlife interactions and potential conflicts [51].

### Mapping of scenario storylines

We used participatory mapping to represent rewilding actions spatially, analyse their distribution, and reveal key trade-offs and synergies [52]. Heat map analysis of the three scenarios highlighted areas with strong stakeholder consensus for implementing specific rewilding strategies, providing valuable insights for future land management in the Oder Delta.

The mapping results indicate that 48% of the identified rewilding areas align with a “Nature for Nature" (NfN) perspective. It corresponds with regional management plans that aim to transition large patches of forested land from commercial forestry to protected areas, abandoned sites, or reduced timber harvesting to facilitate the development of old-growth forests over the next 40 years. Some privately owned areas have already been designated as pilot sites, undergoing gradual reductions in forest management to support this transition [32].

Smaller and more dispersed areas were allocated exclusively for “Nature as Culture" (NaC) scenarios, creating opportunities for biodiversity-friendly agriculture and biocultural conservation. Actions such as peatland rewetting were proposed to support smallholder farming while restoring natural hydrological processes [20,21]. For example, stakeholders identified priority areas for paludiculture—agricultural practices on rewetted peat soils that minimise environmental impact while enabling peat restoration. Biomass harvested from native wetland vegetation, such as reeds and sedges, can serve as a raw material for biofuels, insulation, and construction [53]. This approach supports local livelihoods and enhances long-term ecosystem resilience by maintaining stable water tables and natural disturbance dynamics [54,55]. Beyond direct ecological benefits, rewilding in NaC areas can also help restore human-wildlife relationships [56]. For instance, reducing pressures on forest ecosystems along the German-Polish border could facilitate the return of charismatic species such as bison, elk, and lynx [57].

While certain areas align with a single value perspective, others exhibit overlapping interests where rewilding can support multiple objectives. Transition zones between protected areas, agricultural land, and urban spaces are significant, encompassing diverse ecological, economic, and cultural values [58,59]. However, these areas also present potential land-use conflicts, as multiple stakeholders may have competing interests. Finding a balance between conservation and land use requires measures that integrate ecological restoration with local socio-economic needs. For example, our findings indicate that peatland restoration efforts in areas where all scenarios overlap must balance stakeholder engagement, the recovery of natural water dynamics, and the long-term supply of water resources for consumption. Maintaining natural water levels can enhance ecosystem function and improve the regulation of water resources. However, the successful implementation of these strategies requires collaboration with local communities. It is crucial to involve landowners, offer financial incentives, and integrate rewilding initiatives into local land-use planning to ensure long-term success [60]. Similarly, species reintroduction programs should include compensation schemes and promote non-lethal predator control techniques to secure public support [61,62]. Given the frequent overlap of diverse interests in rewilding contexts, careful and adaptive planning is essential to overcoming potential challenges and achieving optimal outcomes in these initiatives.

Certain areas also present opportunities to integrate conservation with sustainable land management. Overlaps between NaC and NfS scenarios highlight the potential for biodiversity-friendly agricultural practices that enhance food production while improving water quality and climate resilience [53,60]. Rewilding actions that promote the co-production of ecosystem services, such as flood mitigation and drought resilience, can help mitigate the impacts of climate change while maintaining ecosystem integrity [4,63,64].

As highlighted by the stakeholders during the interviews, one promising approach in the Oder Delta is the adoption of paludiculture, which cultivates wet-tolerant plants on rewetted peatlands. This method generates economic benefits through biomass production while simultaneously improving water purification, nutrient retention, and carbon sequestration [46,65]. Furthermore, paludiculture supports peatland-dependent species, contributing to biodiversity conservation [66,67]. The mapping methodology used in this study provides a valuable tool for identifying and anticipating land-use conflicts. By integrating stakeholder perspectives and scenario-based approaches, our framework highlights areas where trade-offs between ecological restoration and socio-economic activities may arise. This enables policymakers to proactively address conflicts and align rewilding initiatives—such as paludiculture—with broader sustainability goals.

### Benefits of participatory scenario planning and assessment for rewilding

The development and assessment of scenarios through participatory processes offers a structured way to integrate diverse stakeholder perspectives into rewilding planning and to better target future interventions across heterogeneous landscapes. By fostering dialogue and identifying trade-offs, participatory approaches improve adaptive decision-making and local acceptance of landscape transformations [22,62], particularly in Europe, where competing land-use demands and conservation priorities require careful consideration [8].

A central finding of this study is that participatory processes are essential for grounding rewilding actions within specific social and ecological contexts. Landscapes are dynamic and heterogeneous, necessitating adaptive management approaches that can respond to diverse contextual demands. Such adaptive management is necessary to address the diverse demands present even within local contexts, such as the Oder Delta, where no single solution is universally applicable. Throughout participatory scenario planning, this heterogeneity was reflected in the identification of rewilding actions tailored to different landscape contexts. For instance, rewetting drained peatlands emerged as a locally relevant priority in areas where disrupted water tables increase drought risk, while transitioning farmers toward paludiculture, supported by targeted training and financial incentives, demonstrated how rewilding can be tailored to align ecological goals with the possibility to improve local livelihoods [68].

Beyond spatial targeting, participatory processes play a proactive role in conflict prevention. The stakeholder interviews revealed that early community involvement is essential for mitigating disputes over resource use and land management, particularly for species reintroductions such as bison and elk, where education on ecological benefits and non-lethal coexistence strategies can reduce human-wildlife conflicts and build long-term support [69,70]. Unlike approaches that address conflict only after it arises [71,72], early stakeholder involvement leads to better perceptions of rewilding initiatives and greater willingness to collaborate [73,74], enabling communities to anticipate challenges and co-develop solutions.

Participatory mapping further strengthens planning by identifying convergence areas across scenarios where high-impact actions can enhance ecological connectivity while aligning with regional conservation strategies [75,76]. By purposively selecting stakeholders who represent key actors in biodiversity conservation decision-making, we ensured that the perspectives gathered reflected the real dynamics of local governance [77,78]. This approach facilitated in-depth dialogue and enabled the collection of reliable, actionable information at early project stages through the combined use of participatory mapping and respondent-driven sampling, ultimately making data collection both cost- and time-efficient [72–74].

Our findings highlight three interconnected benefits of integrating participatory processes into rewilding planning: improved targeting of actions across heterogeneous landscapes; proactive conflict prevention; and stronger collaborative relationships for long-term stewardship. Our methodological framework, integrating participatory scenario planning within the Nature Futures Framework, offers a replicable tool for mapping scenario narratives and associated actions across complex socio-ecological systems [79,80]. Evidence supports that clear communication and active engagement are key drivers of acceptance — when rewilding scenarios were presented as concrete options, broad public willingness to pay for interventions was documented in the Oder Delta, including strong support for large mammal reintroductions [81] and community familiarity with rewilding practices has been shown to markedly increase acceptance across European sites [51]. At the conclusion of the project, we shared the results of the participatory processes with Oder Delta team officials and held focused discussions on their practical application. Officials emphasised that these findings enabled them to reassess priorities, identify areas requiring further community engagement, and outline concrete steps to implement rewilding measures that reflect local realities. Ultimately, our framework offers a practical guide for setting rewilding priorities, fostering meaningful community involvement, and translating rewilding strategies into effective on-the-ground actions.

## Supporting information

S1 Table Detailed scenario storylines and narratives. (PDF)

S2 Table Participatory scenario interview protocol, outlining the structured approach, key questions, and steps used. (PDF)

S3 Table Overview of stakeholders from the net-map analysis, detailing criteria for selection, roles, and interview participation. (PDF)

S1 Fig Template used during participatory mapping exercises with stakeholders, illustrating the format and spatial elements provided for stakeholder input. (PDF)

S2 Fig Network map showing information flow among biodiversity conservation stakeholders in the Oder Delta. Darker arrows show greater impact on decision-making; lighter arrows show lower priority.
(PDF)
