# Supplementary material for: Finding space for rewilding: Nature futures scenarios reveal ecological opportunities based on plural values of nature from participatory processes
Source: PLoS One. 2026 Jul 8;21(7):e0351326. doi: 10.1371/journal.pone.0351326 (PMC13345287; doi:10.1371/journal.pone.0351326)
Supplement: S1 Table — (PDF) [file pone.0351326.s001.pdf]

Table S1. Detailed scenario storylines and narratives.

Nature for Nature- Rewilding for Nature Comeback	Nature for Society - Rewilding for maximising Nature Contributions to People	Nature as Culture*:* Rewilding living in harmony with nature
The rewilding actions focus on restoring lost species interactions and ecosystem functions, aiming to reduce landscape management and preserve nature's diversity and functions, thereby enabling the restoration of self-sufficient and intricate ecosystems. This is achieved by expanding protected areas with stricter protection measures and promoting programmes that facilitate the rehabilitation of keystone species, such as nesting initiatives, thus enhancing trophic complexity. Connectivity is improved by creating green and blue corridors that link protected and high-biodiversity-value areas, while also allowing the natural succession of vegetation in old-growth forests and abandoned areas. Finally, to restore stochastic disturbances, the rewilding actions focus on rewetting dried peatlands to foster biodiversity conservation and restoration.	Enhance the benefits of nature by implementing sustainable management practices that promote biodiversity, a natural capital-based approach, and active management of natural resources. In this scenario, the rewilding actions are centred on providing and restoring various Nature Contributions to People by increasing trophic complexity through the reintroduction of key species, such as wolves and insects, which can provide essential ecosystem services such as pest regulation through predation and pollination. Furthermore, connectivity was promoted through the sustainable use of natural resources in forests and grasslands by decreasing management intensity, thus improving connectivity and creating buffer zones between protected areas and other land uses. Finally, the restoration of natural disturbances focused on providing regulatory services to reduce the risk of flooding and improve the quantity and quality of water and soil through the restoration of water dynamics in abandoned peatlands.	Prioritising community-based management of natural resources, lifestyle changes, and education. This scenario promotes local identities and landscape stewardship by designing rewilding actions that reflect these values. The restoration of trophic complexity aimed to bring back emblematic species (e.g., bison, elk, and grey seal) that can foster local identities. Connectivity of landscapes is promoted to create a rich yet heterogeneous landscape that encourages diverse biodiversity-friendly practices, enabling species to overcome natural barriers. Finally, the promotion of natural disturbances was achieved through community-based rewetting management. An adaptive management approach was followed, with seasonal reflooding of peat soils and grasslands. In areas with high potential for peatland restoration, biodiversity-friendly practices were promoted. Overall, this scenario emphasises the need for community-based management and highlights the cultural and social dimensions of rewilding.
