# Supplementary material for: Finding space for rewilding: Nature futures scenarios reveal ecological opportunities based on plural values of nature from participatory processes
Source: PLoS One. 2026 Jul 8;21(7):e0351326. doi: 10.1371/journal.pone.0351326 (PMC13345287; doi:10.1371/journal.pone.0351326)
Supplement: S2 Table — (PDF) [file pone.0351326.s002.pdf]

Table S2. Participatory scenario interview protocol, outlining the structured approach, key questions, and steps used.

	Step by step guide
**Main objectives for the interview approach**	To gather spatially explicit information about the changes in the landscape associated with different scenarios – participatory mapping.<br>Identification of co-benefits and trade-offs of different NFF scenarios.
**Interview approach**	Interviewers are the principal researchers of the project<br>The aim is to conduct about 10 interviews, each lasting about 1 hour.<br>During this time, one interviewer will ask the questions and lead the interviews, while the other takes notes and visualises results.<br>The interviews will be:<br>• semi-structured, qualitative interviews<br>• held online<br>• audio recorded, and additionally, detailed field notes will be taken
**Leading questions:**	1) Where and how would the three scenarios change the landscape?<br>2) What preferences do interview partners have concerning scenario(s) or individual restoration/rewilding activities?<br>3) Which co-benefits can be related to the three nature futures scenarios?<br>4) Which trade-offs can be related to the three nature futures scenarios?<br>If there is time, the following questions can be covered:<br>5) What could be supporting policies or instruments to realise scenarios or rewilding activities while at the same time fostering co-benefits?
**Interview guideline**	**<u>Phase 1: Introduction & building rapport</u>**<br>• Brief introduction of how the interview will proceed<br>• Have interview partners agree to the consent form and start recording<br>• Short round of introductions:<br>- Principal researcher introduction with the goal and approach of her PhD thesis: developing scenarios for nature restoration and conservation, and models to assess possible impacts of the different scenarios on changes in land use, biodiversity, and ecosystem services.<br>- Introduction of translator and consultant.<br>- Interviewee briefly introduces themselves (Description of their background and projects associated with the Oder Delta)<br>• Brief introduction to the objective of the interview:<br>We are presenting three conservation/restoration scenarios to you at once. We would like to ask you to provide spatially explicit information on the landscape changes associated with the different scenarios. For this purpose, we will show you a map of Uckermünde Heide on which you can draw the changes. It is important that, when assigning changes to the landscape, stakeholders imagine what could happen in 30 years and push away from current political conditions.<br>• Introduce the three scenarios and allow questions of clarification (pay attention to not start to discuss the scenarios, but only give clarifications!)
	**<u>Phase 2: Introduction of the Scenarios:</u>**<br>Presentation of the three scenarios:<br>Scenario 1:<br>This scenario is centred on the creation or expansion of already existing protected areas. The delimitation of protected areas follows stricter regulations, where the intensity of management is reduced and extraction of natural resources is only allowed outside the protected areas. This fosters biodiversity conservation and the restoration of ecosystem functions. The main actions in this scenario are the prioritisation of areas for restoring old-growth forest to foster the recovery of species (and nesting programmes), the rewetting of grasslands and peatlands with high potential for biodiversity restoration, and the creation of green corridors to connect fragmented patches of protected areas.<br>Scenario 2:<br>Nature reserves are expanded or newly created in this scenario. However, more land uses are allowed in the protected areas; for example, sustainable logging and different forms of organic farming may take place around the protected areas. Species that play an important role in pollination and control of pests are prioritised when restoring the landscape. Additionally, rewetting takes place along rivers and forests to restore the natural floodplains.<br>Scenario 3:

	In this scenario, local farmers are encouraged to transition towards more environmentally- and biodiversity-friendly practices that foster rich and diverse agricultural ecosystems. The natural protected areas remain the same in this scenario. The reinforcement of local populations of the grey seal, the lesser spotted eagle, and the elk helps the local community to create a strong link with the identity of the area, thus making these species long-term emblems of the Oder Delta. Finally, the rewetting of grasslands and peatlands only takes place where there is high potential for biodiversity restoration and where it does not result in the loss of grasslands for productive purposes.
	**<u>Phase 3: Participatory Mapping</u>**<br>The interviewees will be presented with a map that has information on the current land uses of the area (i.e., protected areas, peatlands, moors, grasslands and agricultural sites). They will be asked to indicate whether they think the area of the existing land use category can increase or whether a new land use area should be created in the next 10-30 years. It is important to highlight that we want information at the local level.<br>• The mapping exercise will be done for each scenario separately. However, based on previous tests, we group the questions per scenario into the following categories:<br>1) Increase or expansion of protected areas?<br>2) Where organic agriculture or forms of sustainable agriculture could take place<br>3) Rewetting of peatlands and moors<br>4) Creation of green corridors<br>• Before starting the interview, it is important to contextualise the stakeholders: first, we want information at the local level, and second, we will tell them to imagine changes while ignoring current land ownerships.<br><br>Start of the participatory mapping process:<br><br>1) <u>Protected areas/ecological agriculture.</u><br><br>In two of the three scenarios, protected areas would be expanded or newly created into two categories: in scenario 1, land use (agriculture and forestry) would be stopped, but people could still go there (e.g., for tourism). In scenario 2, organic agriculture would be permitted in protected areas. Where would agriculture take place in these areas?<br>Where would they draw in the protected areas in the first two scenarios (different areas or the same areas)?<br>In the third scenario, no protected areas would be expanded, but people would voluntarily adopt organic farming practices. Imagine that half of the agricultural land were organic. Where would this land be most usefully placed for biodiversity?<br>In scenario 1:<br>Q1a: Where would Strict protected areas be expanded or newly designated?<br>Q1b: Where would old, unused forests increase?<br>In scenario 2:<br>Q2: Where could organic agriculture or sustainable logging increase in the protected areas? Or outside?<br>In scenario 3:<br>Q3: 50% of Cultivated areas transition towards organic agriculture. Where would this land be most usefully placed for biodiversity?<br>2) Rewetting:<br>Across all three scenarios, rewetting should occur but for different reasons.<br>In scenario 1<br>Q1: Rewetting areas promotes biodiversity and reinforces local populations. Where would this happen in the map?<br>In scenario 2<br>Q2: Rewetting of forest, peatlands and river beds to create natural floodplains and improve the regulation of water dynamics of the area. Where would this happen in the map?<br>In scenario 3<br>Q3: The goal is to lose as little of the productive grassland as possible during rewetting. Where would this happen in the map?<br>Do you see differences in the potential for rewetting on the map for the different reasons?<br>3) Green corridors<br>Q1: In Scenario 1, where on the map do you see opportunities to create ecological corridors? (explanation of green corridors: to connect separated patches of nature reserves)

	**Phase 4: Scenario preferences**<br>• After mapping, the stakeholder will be asked to state their preferences towards the three scenarios<br>• If there is no explicit preference, they are allowed to create a 4th scenario with the components they like the most from each scenario.<br>Q1: Would you have a preference for a particular scenario, or which scenario would be the least desirable?
	**Phase 5: Co-benefits and trade offs**<br>• After mapping, the stakeholder will be asked to name the main co-benefits for each scenario, and if the different scenarios share co-benefits or trade-offs<br>• It is important to give stakeholders a short explanation of what the co-benefits and trade-offs mean. (Note: We should give the same example to all interviewees.)<br>Q1a: Which co-benefits can be related to the different scenarios?<br>Q1b: Are there co-benefits shared between the scenarios?<br>Q2a: Which challenges can be related to the different scenarios?<br>Q2b: Are there challenges shared between the scenarios?<br>The answers will be recorded and noted by the interviewers.
	**Phase 6: Wrap up**<br>Ask the interview partner whether he/she has any additional questions or comments.<br>Ask interview partners whom we should additionally interview.<br>Ask whether they are interested in staying in contact with the project and in seeing the outcomes of the interviews.<br>Thank the interview partners and end the interview.
**Documentation**	Documentation using online whiteboards; Audio recordings, detailed field notes, post script (the two interviewers meet immediately after the meeting and share their most important observations)
**Data analysis**	Systeatic Qualitative content analysis, basic descriptive statistics.
