# Supplementary material for: Finding space for rewilding: Nature futures scenarios reveal ecological opportunities based on plural values of nature from participatory processes
Source: PLoS One. 2026 Jul 8;21(7):e0351326. doi: 10.1371/journal.pone.0351326 (PMC13345287; doi:10.1371/journal.pone.0351326)
Supplement: S3 Table — (PDF) [file pone.0351326.s003.pdf]

Table S3. Overview of stakeholders from the net-map analysis, detailing criteria for selection, roles, and interview participation.

Netmap Relevance (arrows)	Key information flows (darker arrows)	Stakeholder Group	Working area	Contacted	Interviewed
12;3	8;1	Conservation NGO, Civil society association	Biodiversity conservation, Eco-tourism	YES	YES
9	5	Conservation NGO	forest conservation	YES	YES
8	7	Public Administration	Tourism -biodiversity conservation-land management	YES	YES
8	4	Conservation NGO	Biodiversity Conservation	YES	YES
7	2	Public Administration	Biodiversity Conservation	YES	YES
7	2	Public Administration	Biodiversity Conservation	YES	YES
4;2	1;0	Conservation NGO; Academia	Water Management	YES	YES
4	0	Public Administration	Water Management	YES	NO
4	1	Conservation NGO	Water Management	YES	NO
3	3	Civil society association	Sustainable Agriculture sector	YES	YES
3 ; 2	3 ; 0	Civil Society association	Forestry - Hunter Sector	YES	YES
2	0	Civil society association	Hunting Sector	YES	NO
2	0	Civil society association	Hunter Sector	YES	NO
1	0	Academia	Academia	NO	NO
1	0	Civil society associaton	Journalism	YES	NO
0	0	Civil society association	Agriculture sector	NO	NO
