# Supplementary material for: Finding space for rewilding: Nature futures scenarios reveal ecological opportunities based on plural values of nature from participatory processes
Source: PLoS One. 2026 Jul 8;21(7):e0351326. doi: 10.1371/journal.pone.0351326 (PMC13345287; doi:10.1371/journal.pone.0351326)

Figure S1. Template used during participatory mapping exercises with stakeholders, illustrating the format and spatial elements provided for stakeholder input.

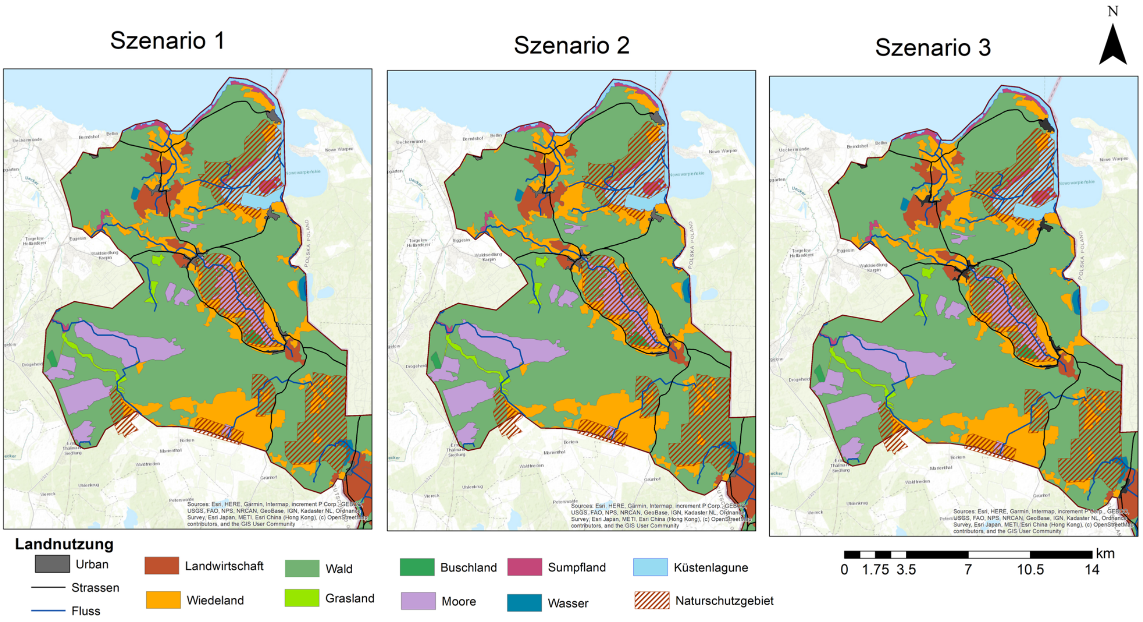

Supplement: S1 Fig — (PDF) [file pone.0351326.s004.pdf]
