# Supplementary material for: Finding space for rewilding: Nature futures scenarios reveal ecological opportunities based on plural values of nature from participatory processes
Source: PLoS One. 2026 Jul 8;21(7):e0351326. doi: 10.1371/journal.pone.0351326 (PMC13345287; doi:10.1371/journal.pone.0351326)

Figure S2. Network map showing information flow among biodiversity conservation stakeholders in the Oder Delta. Darker arrows show greater impact on decision-making; lighter arrows show lower priority.

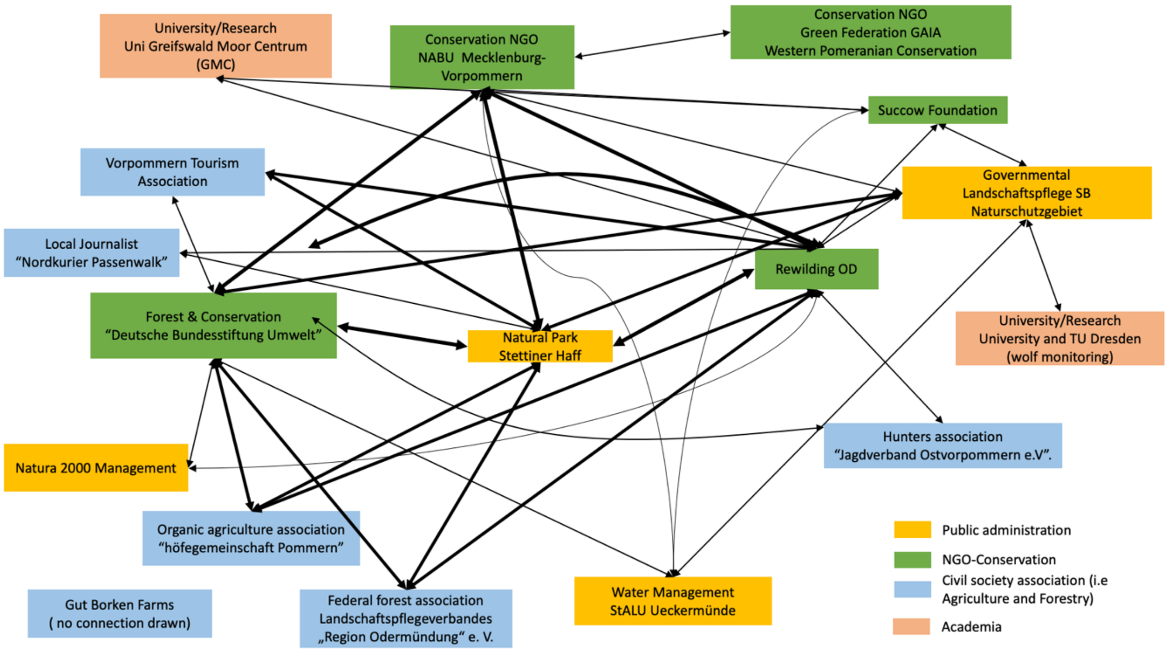

Supplement: S2 Fig — Darker arrows show greater impact on decision-making; lighter arrows show lower priority. (PDF) [file pone.0351326.s005.pdf]
