# Supplementary material for: Finding space for rewilding: Nature futures scenarios reveal ecological opportunities based on plural values of nature from participatory processes
Source: PLoS One. 2026 Jul 8;21(7):e0351326. doi: 10.1371/journal.pone.0351326 (PMC13345287; doi:10.1371/journal.pone.0351326)
Supplement: Attachment — Submitted filename: response reviews PONE-D-25-51781.docx [file pone.0351326.s007.docx]

**Cover Letter / Response to Reviewers**

**Manuscript ID** PONE-D-25-51781

**Title:** Finding Space for Rewilding: Nature Futures Scenarios Reveal Ecological Opportunities Based on Plural Values of Nature from Participatory Processes

Dear Dr Miguel Inácio,

We thank you for the opportunity to revise our manuscript and appreciate the constructive feedback from the reviewers, which helped strengthen our methodology and the clarity of the manuscript.

We have addressed all comments raised by the Academic Editor and the reviewers. Below is a point-by-point response detailing how each issue has been resolved.

**Response to Academic Editor / Journal Requirements**

**1. PLOS ONE Style Requirements**

*Please ensure that your manuscript meets PLOS ONE's style requirements, including those for file naming.*

**Response:** We have carefully reviewed the PLOS ONE style templates and formatted the manuscript, title page, and file names accordingly.

**2.Funding Statement and Financial Disclosure**

*Please provide an amended statement that declares all the funding... Please state what role the funders took... Please remove any funding-related text from the manuscript's Acknowledgements.*

**Response:**

- Thanks for noting the repetition of the information. We have now  removed the funding information from the Acknowledgements section of the manuscript.
- The updated and corrected funding statement is the following:  “L.Q-U., N.F., H.P., and R.D-C. gratefully acknowledge the support of the German Centre for Integrative Biodiversity Research (iDiv) Halle-Jena-Leipzig, funded by the German Research Foundation (DFG) (DFG–FZT 118, 202548816; https://www.idiv.de/). L.Q-U. was supported by the European Union’s Horizon 2020 research and innovation programme under the Marie Skłodowska-Curie grant agreement No 813904 (https://marie-sklodowska-curie-actions.ec.europa.eu/), and by the project REWILD_DE-2 (funding code 16LW0064K) within the Research Initiative for the Conservation of Biodiversity (FEdA), funded by the German Federal Ministry of Education and Research (BMBF) (https://www.feda.bio/en/). J-S received no specific funding for this work. The funders had no role in study design, data collection and analysis, decision to publish, or preparation of the manuscript. There was no additional external funding received for this study.”

**3. Title Page**

*Please ensure that you include a title page within your main document.*

**Response:** A title page including all authors, affiliations, and the corresponding author has been added to the main document.

**6. Supporting Information Captions**

*Please include captions for your Supporting Information files at the end of your manuscript.*

**Response:** We have added a list of captions for all Supporting Information files at the end of the manuscript.

**7. Copyrighted Map Figures (Figures 1, 3, & 4)**

*We note that Figures 1, 3 & 4 in your submission contain [map/satellite] images which may be copyrighted... We require you to either (1) present written permission... or (2) remove the figures.*

**Response:** We have now carefully checked the licensing of the map layers used in Figures 1, 4, and 5 (formerly Figures 1, 3, and 4). The layers included in these figures and their copyright permissions are as follows:

1. Copernicus (Land Use Data): Fully compatible with CC BY 4.0.

2. Eurostat / GISCO (Administrative Boundaries): CC BY 4.0.

3. Protected Areas: We replaced the previous layers from LUNG M-V (which were only available under CC BY-SA) with the European inventory of Nationally Designated Areas, licensed under CC BY 4.0 (https://creativecommons.org/licenses/by/4.0/). The copyright holder is the European Environment Agency (EEA).

All map layers used in these figures are now fully compatible with the CC BY 4.0 license required by the journal.

**Reviewer Comments:**

**Reviewer #1**

1. *Figure S1 illustrates very well the steps applied in the study, therefore I would suggest including it in the main part of the text.* **Response:** We agree with this suggestion. We have moved Figure S1 into the main text; it is now labelled as **Figure** 2
2. *Figure S3 is referred in the text as “S2 Figure”.* **Response:** Thank you for spotting this error. We have corrected the citation in the text to refer to the correct figure.
3. *Caption of Figure 2: Perhaps the sentence “The circles represent how often specific actions were mentioned in different scenarios” could be removed since the next sentence repeats the same information.* **Response:** We have removed the redundant sentence from the figure caption. It now states as follows: “ **Figure 2. Frequency of main actions mentioned by the stakeholders for the different NFF scenarios.** Circle size and colour indicate the frequency with which specific actions were mentioned across scenarios: smaller red circles correspond to less frequently mentioned actions, while larger, darker blue circles represent actions cited more often.”
4. *Line 386: “The distribution of the three scenarios covered only 1.91%” – does this refer to the overlap between the three scenarios?* **Response:** Yes, this refers to the overlap. We have rephrased this sentence to make it clearer as follows: “ The regions where all three scenarios spatially overlapped on the map covered only 1.91% of the total study area. These areas were found  adjacent to natural parks, extensive livestock grasslands, and urban settlements.”
5. *Lines 536-537: “The research method demonstrated in this study aided in identifying” - it looks like this sentence is incomplete.* **Response:** Thanks for noting this issue. We have completed the sentence and also improved the readability of the text. This new information can be found on lines 817-826*.*
6. *Discussion: ...Perhaps the authors could elaborate on whether there is any evidence about the acceptance of rewilding among the public and farmers and what actions would be necessary to promote the implementation...* **Response:** We have expanded the Discussion section to address both public and farmer acceptance. The new information can be found in lines 851–862. Specifically, we refer to previous studies from the region that examine acceptance of rewilding and willingness to pay, as well as research highlighting the importance of public education in improving acceptance before implementing rewilding actions. Additionally, we include our reflections on interactions with the rewilding Oder Delta team during the presentation and discussion of outcomes from the participatory process.

**Reviewer #2**

1. *My main criticism concerning the manuscript is that the methodology remains rather abstract... I found the manuscript to be very difficult to follow and understand.* **Response:** We thank the reviewer for highlighting this important issue. We appreciate this feedback and, as a result, have substantially revised the Methodology section to provide greater detail and transparency. The revised text now elaborates on the sequence and structure of the participatory processes, providing clearer descriptions of each methodological step. We have included an expanded explanation of the Net-Map tool, its protocol, and how it was implemented in our study (see lines 200–217). This new content clarifies the rationale for selecting Net-Map, details the steps undertaken during its application, and outlines how the resulting network data were analysed and integrated into subsequent phases of the research. We have also clarified the distinct roles of the stakeholder groups involved in scenario planning and assessment (see lines 177-184), and have incorporated this updated information into Figure 2. These changes make the methodology section clearer and easier to follow, with a full account of our tools and procedures.
2. *The local context and need for rewilding the German Oder Delta remain unclear. Furthermore, it should be explained why changes over 30 years are considered in the study area.* **Response:** We have added a new paragraph to the Introduction (lines 114–123) and expanded the Study Area description (lines 142–170) to explain the region’s ecological importance and the specific need for rewilding initiatives. Additionally, we now provide a clear justification for considering a 30-year timeframe, which is consistent with both the Kunming-Montreal Global Biodiversity Framework and the EU Restoration Law (see lines 243–246).
3. *There is no information about the applied tool or how the resulting map was used in the study... why it is not presented in the results. Response:* We have added a detailed description of the Net-Map tool in the Methodology section and information on how and for what we used the mapping results.
4. *A “Net-Map tool” was applied identify stakeholder groups. The resulting network map is presented in supplements. Yet, there is not information about the applied tool or how the resulting map was used in the study. Its is also not clear, why it is not presented in the results. Furthermore, the involvement of stakeholders in the development of the scenarios storylines is mentioned in the methodology (phase 2) and several times in the discussion (Oder Delta experts are not stakeholders use better wording but emphasize it was a collaborative effort to understand local dynamics and future goals to carry out rewilding in the region). Yet, it is not clear who these stakeholders are and if there is an overlap with the interviewed stakeholders. Were the interviewees involved in developing the scenarios? (NO) Or were the scenarios already completed when having the interviews?* **Response:** Thank you for highlighting this important point. We have now added two paragraphs that explain in detail how the Net-Map tool methods were conducted, including the phases involved in their execution (see lines 192–217). We now include the outcomes of the Net-Map tool in both the Results and Discussion sections and describe how this information informed the next steps of the research. We wish to clarify that, in the first iteration of the manuscript, we decided to include information on the Net-Map tool in the supplementary material, as it is not a main outcome of the research but rather a methodological step to facilitate the scenario assessment. We understand that this could be confusing to readers; therefore, we have expanded the text to further explain the processes and how we used the results. However, we have chosen to keep the full network map in the supplementary material, as the key results and their implications are now explained in the Results section, and readers seeking more detailed information can refer to the supplementary files. We believe this approach is appropriate, as the Net-Map is a crucial step in presenting our framework for participatory scenario development, but it is not the main outcome or narrative of the article. Additionally, we provided further clarification regarding the stakeholder groups. Specifically, we clarify that the stakeholders interviewed for the Net-Map analysis were distinct from those selected to assess the already defined scenario storylines. There was no overlap between the two groups. The first group of stakeholders collaborated with us to develop the initial storylines, while the second group participated in assessing and evaluating these storylines through participatory processes. These clarifications improve transparency regarding stakeholder involvement (Lines 177-184 and Figure 2).
5. *It is a little confusing that the authors mention four steps in the method, but then refer to a Figure that shows 5 steps.* **Response:** Thanks for noticing this inconsistency. We have aligned the text and the figure. The text now accurately describes the 5 steps shown in the figure (see lines 182-184)
6. *Please clarify the terms Narratives, Scenarios, scenario storylines, Actions and Key themes?* **Response:**Thank you for the suggestion. We have added a section in the methods of “Terminology and Definitionsto” clarify the vocabulary used throughout the manuscript ( see lines 177-193)
7. *Please review and correct the scales that are shown in the figures.* **Response:** We checked all scale bars from the map figures and they are correct.
8. *“S2 Figure” (referred to in Line 159) is missing from the supplementary material.* **Response:**Thank you for noticing this issue. Figure S2 was mistakenly labelled as S3; we have now corrected this error.
9. *Lines 237-238: maps seem to be lacking from the S5 Table.***Response:** We added the map templates used during the interviews; they can be found in Supplementary material SX.
10. *Word missing in the caption of Fig. 4.***Response:** We have corrected the caption for Figure 4.The new captions state as follows: “**Consolidated map illustrating the spatial distribution of areas designated for biodiversity conservation, rewetting, blue-green corridors, and biodiversity-friendly agriculture across various scenario narratives.** The colours represent each Nature Future Scenario: green for “Nature for Nature,” blue for “Nature for Society,” and yellow for “Nature as Culture.” Darker shades indicate areas identified by 8 or 9 stakeholders, while lighter shades represent areas identified by 1 or 2 stakeholders. **B. Map illustrating the intersection of the three scenario maps found in section A.** Areas are categorised based on their degree of spatial or physical overlap among the three scenarios, showing distinct zones associated with only one scenario, areas of intersection between two scenarios, and regions where all three scenarios converge.”
11. *Meaning of different colours 3 and 4A should be explained.* **Response:** We have added a legend/explanation for the colours in the caption of Figures 3 and 4A. (see lines  499 - 502 and 508-510)
12. *Be consistent in the use of American or British English.***Response:** Thanks for noticing this issue. We have standardised the manuscript to British English.
13. *L398: correct “stakeholders” / L403 – correct “on a large scale studies”.***Response:** Thanks for noticing these errors; they have now been corrected.
