# Supplementary material for: Finding space for rewilding: Nature futures scenarios reveal ecological opportunities based on plural values of nature from participatory processes
Source: PLoS One. 2026 Jul 8;21(7):e0351326. doi: 10.1371/journal.pone.0351326 (PMC13345287; doi:10.1371/journal.pone.0351326)
Supplement: Attachment — Submitted filename: response_reviews_PONE-D-25-51781_auresp_2.docx [file pone.0351326.s008.docx]

**Response to Reviewers**

**Manuscript ID** PONE-D-25-51781

**Title:** Finding Space for Rewilding: Nature Futures Scenarios Reveal Ecological Opportunities Based on Plural Values of Nature from Participatory Processes

Dear Dr Miguel Inácio,

We truly appreciate the thoughtful and constructive feedback from you and the reviewers on our revised manuscript. We have updated the abstract to better reflect the substantial changes made during both the first review and this revision, condensing all main points into a single, cohesive paragraph. We have carefully addressed all comments to make the manuscript as clear and precise as possible. Please find our point-by-point responses below.

**Comments Reviewer #1**

**1.** *"I acknowledge that the manuscript has been significantly improved, and I would like to thank the authors for carefully considering the reviewers’ comments. However, before publication, I would recommend that the authors carefully review the text to avoid repetitions in both phrasing and content, particularly in the introduction."****. Response:*** *Thank you for highlighting this important aspect. We have now thoroughly reviewed the manuscript, with particular attention to the introduction. We removed redundant phrasing and repeated concepts, streamlined the flow of ideas to enhance clarity and conciseness, and ensured that each concept is introduced and developed only once, avoiding unnecessary repetition. These changes are mainly reflected in L42-46; L68-71; L73-95; L110-122.*

**Comments to Reviewer #2**

1. Line 200: Grammar correction *"...which consisted of two".* ***Response****: The text has been corrected as suggested.*
2. Line 379: Typo ("Deltaand"). ***Response****: Fixed to "Delta and".*
3. Line 380: "Strength" vs. "strengths". ***Response****: Revised to "strengths" for grammatical accuracy.*
4. Table 2: Clarification of notation (e.g., 12;3 and 8;1). ***Response****: We have added a footnote to Table 2 to explain the format as follows: “* two numbers indicate two different actors, each with their own values for mapped relationships and relevance”.*

We believe these changes have enhanced the clarity and overall quality of the article.

Best regards,

Laura C. Quintero Uribe, on behalf of the coauthors.
