# Supplementary material for: Finding space for rewilding: Nature futures scenarios reveal ecological opportunities based on plural values of nature from participatory processes
Source: PLoS One. 2026 Jul 8;21(7):e0351326. doi: 10.1371/journal.pone.0351326 (PMC13345287; doi:10.1371/journal.pone.0351326)
Supplement: Peer review history

# Peer review: Decision Letter 0

**DOI:** 10.1371/journal.pone.0351326.r001

**Authors:** Miguel Inácio

30 Dec 2025

PONE-D-25-51781

Finding Space for Rewilding: Nature Futures Scenarios Reveal Ecological Opportunities Based on Plural Values of Nature from Participatory Processes

PLOS One

Dear Dr. Quintero Uribe,

Thank you for submitting your manuscript to PLOS ONE. After careful consideration, we feel that it has merit but does not fully meet PLOS ONE’s publication criteria as it currently stands. Therefore, we invite you to submit a revised version of the manuscript that addresses the points raised during the review process.

==============================

**Dear authors,**

**Thank you for submitting your manuscript to PLOS One. Based on the comments of both reviewers there are several critical aspects that need to be addressed. Please refer to all comments and suggestions by the reviewers.**

==============================

Please submit your revised manuscript by Feb 13 2026 11:59PM. If you will need more time than this to complete your revisions, please reply to this message or contact the journal office at plosone@plos.org. When you're ready to submit your revision, log on to https://www.editorialmanager.com/pone/ and select the 'Submissions Needing Revision' folder to locate your manuscript file.

Please include the following items when submitting your revised manuscript:

- A letter that responds to each point raised by the academic editor and reviewer(s). You should upload this letter as a separate file labeled 'Response to Reviewers'.
- A marked-up copy of your manuscript that highlights changes made to the original version. You should upload this as a separate file labeled 'Revised Manuscript with Track Changes'.
- An unmarked version of your revised paper without tracked changes. You should upload this as a separate file labeled 'Manuscript'.

If you would like to make changes to your financial disclosure, please include your updated statement in your cover letter. Guidelines for resubmitting your figure files are available below the reviewer comments at the end of this letter.

If applicable, we recommend that you deposit your laboratory protocols in protocols.io to enhance the reproducibility of your results. Protocols.io assigns your protocol its own identifier (DOI) so that it can be cited independently in the future. For instructions see: https://journals.plos.org/plosone/s/submission-guidelines#loc-laboratory-protocols. Additionally, PLOS ONE offers an option for publishing peer-reviewed Lab Protocol articles, which describe protocols hosted on protocols.io. Read more information on sharing protocols at https://plos.org/protocols?utm_medium=editorial-email&utm_source=authorletters&utm_campaign=protocols.

We look forward to receiving your revised manuscript.

Kind regards,

Miguel Inácio

Academic Editor

PLOS One

Journal Requirements:

When submitting your revision, we need you to address these additional requirements.

1. Please ensure that your manuscript meets PLOS ONE's style requirements, including those for file naming. The PLOS ONE style templates can be found at

https://journals.plos.org/plosone/s/file?id=wjVg/PLOSOne_formatting_sample_main_body.pdf and

https://journals.plos.org/plosone/s/file?id=ba62/PLOSOne_formatting_sample_title_authors_affiliations.pdf.

2. Thank you for stating in your Funding Statement:

“The authors gratefully acknowledge the support of iDiv funded by the German Research Foundation (DFG-FZT 118, 202548816). LQ-U was supported by funding from the European Union's Horizon 2020 Programme “Marie Sklodowska-Curie Innovative Training Networks” (MSCA-ITN-ETN, grant no. 813904), and from the FEdA programme of the Federal Ministry of Education and Research of Germany (BMBF) (grant no. 16LW0064K).”

Please provide an amended statement that declares *all* the funding or sources of support (whether external or internal to your organization) received during this study, as detailed online in our guide for authors at http://journals.plos.org/plosone/s/submit-now.  Please also include the statement “There was no additional external funding received for this study.” in your updated Funding Statement.

Please include your amended Funding Statement within your cover letter. We will change the online submission form on your behalf.

3. Thank you for stating the following financial disclosure:

“The authors gratefully acknowledge the support of iDiv funded by the German Research Foundation (DFG-FZT 118, 202548816). LQ-U was supported by funding from the European Union's Horizon 2020 Programme “Marie Sklodowska-Curie Innovative Training Networks” (MSCA-ITN-ETN, grant no. 813904), and from the FEdA programme of the Federal Ministry of Education and Research of Germany (BMBF) (grant no. 16LW0064K).”

Please state what role the funders took in the study.  If the funders had no role, please state: "The funders had no role in study design, data collection and analysis, decision to publish, or preparation of the manuscript."

If this statement is not correct you must amend it as needed.

Please include this amended Role of Funder statement in your cover letter; we will change the online submission form on your behalf.

4. Thank you for stating the following in the Acknowledgments Section of your manuscript:

“The authors gratefully acknowledge the support of iDiv funded by the German Research Foundation (DFG-FZT 118, 202548816). LQ-U was supported by funding from the European Union's Horizon 2020 Programme “Marie Sklodowska-Curie Innovative Training Networks” (MSCA-ITN-ETN, grant no. 813904), and from the FEdA programme of the Federal Ministry of Education and Research of Germany (BMBF) (grant no. 16LW0064K).”

We note that you have provided additional information within the Acknowledgements Section that is not currently declared in your Funding Statement. Please note that funding information should not appear in the Acknowledgments section or other areas of your manuscript. We will only publish funding information present in the Funding Statement section of the online submission form.

Please remove any funding-related text from the manuscript and let us know how you would like to update your Funding Statement. Currently, your Funding Statement reads as follows:

“The authors gratefully acknowledge the support of iDiv funded by the German Research Foundation (DFG-FZT 118, 202548816). LQ-U was supported by funding from the European Union's Horizon 2020 Programme “Marie Sklodowska-Curie Innovative Training Networks” (MSCA-ITN-ETN, grant no. 813904), and from the FEdA programme of the Federal Ministry of Education and Research of Germany (BMBF) (grant no. 16LW0064K).”

Please include your amended statements within your cover letter; we will change the online submission form on your behalf.

5. Please ensure that you include a title page within your main document. You should list all authors and all affiliations as per our author instructions and clearly indicate the corresponding author.

6. Please include captions for your Supporting Information files at the end of your manuscript, and update any in-text citations to match accordingly. Please see our Supporting Information guidelines for more information: http://journals.plos.org/plosone/s/supporting-information.

7. We note that Figures 1, 3 & 4 in your submission contain [map/satellite] images which may be copyrighted. All PLOS content is published under the Creative Commons Attribution License (CC BY 4.0), which means that the manuscript, images, and Supporting Information files will be freely available online, and any third party is permitted to access, download, copy, distribute, and use these materials in any way, even commercially, with proper attribution. For these reasons, we cannot publish previously copyrighted maps or satellite images created using proprietary data, such as Google software (Google Maps, Street View, and Earth). For more information, see our copyright guidelines: http://journals.plos.org/plosone/s/licenses-and-copyright.

We require you to either (1) present written permission from the copyright holder to publish these figures specifically under the CC BY 4.0 license, or (2) remove the figures from your submission:

a. You may seek permission from the original copyright holder of Figures 1, 3 & 4 to publish the content specifically under the CC BY 4.0 license.

We recommend that you contact the original copyright holder with the Content Permission Form (http://journals.plos.org/plosone/s/file?id=7c09/content-permission-form.pdf) and the following text:

“I request permission for the open-access journal PLOS ONE to publish XXX under the Creative Commons Attribution License (CCAL) CC BY 4.0 (http://creativecommons.org/licenses/by/4.0/). Please be aware that this license allows unrestricted use and distribution, even commercially, by third parties. Please reply and provide explicit written permission to publish XXX under a CC BY license and complete the attached form.”

Please upload the completed Content Permission Form or other proof of granted permissions as an "Other" file with your submission.

In the figure caption of the copyrighted figure, please include the following text: “Reprinted from [ref] under a CC BY license, with permission from [name of publisher], original copyright [original copyright year].”

b. If you are unable to obtain permission from the original copyright holder to publish these figures under the CC BY 4.0 license or if the copyright holder’s requirements are incompatible with the CC BY 4.0 license, please either i) remove the figure or ii) supply a replacement figure that complies with the CC BY 4.0 license. Please check copyright information on all replacement figures and update the figure caption with source information. If applicable, please specify in the figure caption text when a figure is similar but not identical to the original image and is therefore for illustrative purposes only.

The following resources for replacing copyrighted map figures may be helpful:

USGS National Map Viewer (public domain): http://viewer.nationalmap.gov/viewer/

The Gateway to Astronaut Photography of Earth (public domain): http://eol.jsc.nasa.gov/sseop/clickmap/

Maps at the CIA (public domain): https://www.cia.gov/library/publications/the-world-factbook/index.html and https://www.cia.gov/library/publications/cia-maps-publications/index.html

NASA Earth Observatory (public domain): http://earthobservatory.nasa.gov/

Landsat: http://landsat.visibleearth.nasa.gov/

USGS EROS (Earth Resources Observatory and Science (EROS) Center) (public domain): http://eros.usgs.gov/#

Natural Earth (public domain): http://www.naturalearthdata.com/

8. If the reviewer comments include a recommendation to cite specific previously published works, please review and evaluate these publications to determine whether they are relevant and should be cited. There is no requirement to cite these works unless the editor has indicated otherwise.

[Note: HTML markup is below. Please do not edit.]

Reviewers' comments:

Reviewer's Responses to Questions

**Comments to the Author**

1. Is the manuscript technically sound, and do the data support the conclusions?

The manuscript must describe a technically sound piece of scientific research with data that supports the conclusions. Experiments must have been conducted rigorously, with appropriate controls, replication, and sample sizes. The conclusions must be drawn appropriately based on the data presented.

Reviewer #1: Yes

Reviewer #2: Partly

**********

2. Has the statistical analysis been performed appropriately and rigorously?

Reviewer #1: N/A

Reviewer #2: N/A

**********

3. Have the authors made all data underlying the findings in their manuscript fully available?

The PLOS Data policy requires authors to make all data underlying the findings described in their manuscript fully available without restriction, with rare exception (please refer to the Data Availability Statement in the manuscript PDF file). The data should be provided as part of the manuscript or its supporting information, or deposited to a public repository. For example, in addition to summary statistics, the data points behind means, medians and variance measures should be available. If there are restrictions on publicly sharing data—e.g. participant privacy or use of data from a third party—those must be specified.

Reviewer #1: Yes

Reviewer #2: Yes

**********

4. Is the manuscript presented in an intelligible fashion and written in standard English?

PLOS ONE does not copyedit accepted manuscripts, so the language in submitted articles must be clear, correct, and unambiguous. Any typographical or grammatical errors should be corrected at revision, so please note any specific errors here.

Reviewer #1: Yes

Reviewer #2: No

**********

5. Review Comments to the Author

Please use the space provided to explain your answers to the questions above. You may also include additional comments for the author, including concerns about dual publication, research ethics, or publication ethics. (Please upload your review as an attachment if it exceeds 20,000 characters)

Reviewer #1: This paper explores the highly topical issue of rewilding, which is crucial in the context of the global biodiversity crises and international policy commitments (e.g. EU Nature Restoration Law). The study is based on participatory scenario development that reveal pluralistic value perspectives of rewilding activities and opportunities to use local knowledge to identify the possible co-benefits and trade-offs of such practices. The paper is well written and clearly structured, providing a good overview of the conceptual basis of the topic as well as a clear description of the methodology, results and their interpretation in relation to future land use and its planning policy. I consider the paper acceptable for publication in the journal, after addressing few minor points:

1) Figure S1 illustrates very well the steps applied in the study, therefore I would suggest including it in the main part of the text.

2) Figure S3 is referred in the text as “S2 Figure”.

3) Caption of the Figure 2: Perhaps the sentence “The circles represent how often specific actions were mentioned in different scenarios” could be removed since the next sentence repeats the same information.

4) Line 386: “The distribution of the three scenarios covered only 1.91%” – does this refer to the overlap between the three scenarios?

5) Lines 536-537: “The research method demonstrated in this study aided in identifying” - it looks like that this sentence is incomplete.

6) Discussion: Authors highlight that participatory processes of scenario development can support adaptive decision-making and local acceptance of landscape transformations. However, the study involved only nine key stakeholders from agriculture, forestry, nature conservation, and administrative institutions. Perhaps the authors could elaborate on whether there is any evidence about the acceptance of the rewilding among public and farmers and what actions would be necessary to promote the implementation of the proposed restoration measures in practice.

Reviewer #2: This study presents a methodological framework, based on the Nature Futures Framework, for participatory scenario development in rewilding initiatives, applying it to the context of the German Oder Delta — a topic that is certainly worth exploring. However, my main criticism concerning the manuscript is that the methodology remains rather abstract. Thus, I found the manuscript to be very difficult to follow and understand. In addition, the study context and broader scientific relevance are not sufficiently presented.

For instance, the location of the study area and major land uses are introduced. However, the local context and need for rewilding the German Oder Delta remain unclear. Furthermore, it should be explained why changes over 30 years are considered in the study area.

In the methodology, the authors state that the scenario development process consisted of four phases, including (1) participant identification and sampling, (2) development of scenario storylines, (3) semi-structured interviews, and (4) data analysis. Each phase is described in a subchapter of the methodology chapter. However, at several points the authors only refer to the supplementary material, without providing sufficient information in the manuscript. For instance, a “Net-Map tool” was applied identify stakeholder groups. The resulting network map is presented in supplements. Yet, there is not information about the applied tool or how the resulting map was used in the study. Its is also not clear, why it is not presented in the results. Furthermore, the involvement of stakeholders in the development of the scenarios storylines is mentioned in the methodology (phase 2) and several times in the discussion. Yet, it is not clear who these stakeholders are and if there is an overlap with the interviewed stakeholders. Were the interviewees involved in developing the scenarios? Or were the scenarios already completed when having the interviews?

There is a nice Figure in the supplementary material (S1 Fig.) which visualizes the steps. This could be moved to the main text and modified to show who (authors, project participants, stakeholders, etc.) was involved in each step. Yet, it is a little confusing that the authors mention four steps in the method, but then refer to a Figure that shows 5 steps.

Please clarify the terms Narratives, Scenarios, scenario storylines, and Actions and Key themes? It was not clear for me if they partially refer to the the same aspects, or if there is a clear distinction

Some more specific comments:

Figures & Tables

- Please review and correct the scales that are shown in the figures (e.g. Figure 1)

- “S2 Figure” (referred to in Line 159) is missing is the supplementary material

- There are several spelling mistakes in Table S5. Please review and correct

- In Lines 237-238 it is stated that the template and maps used in the stakeholder interviews are provided in S5 Table. However, the maps seem to be lacking.

- There is a word missing in the caption of Fig. 4

- Meaning of different colours 3 and 4A should be explained

Spelling mistakes, etc.

- Carefully check the supplementary material for mistakes

- Be consistent in the use of American or British English

- Spell abbreviations in full, when they occur for the first time (e.g. GDPR)

- L398: correct “stakeholdersr”

- L403 – correct “on a large scale studies”

**********

6. PLOS authors have the option to publish the peer review history of their article (what does this mean?). If published, this will include your full peer review and any attached files.

If you choose “no”, your identity will remain anonymous but your review may still be made public.

**Do you want your identity to be public for this peer review?**  For information about this choice, including consent withdrawal, please see our Privacy Policy.

Reviewer #1: No

Reviewer #2: No

**********

[NOTE: If reviewer comments were submitted as an attachment file, they will be attached to this email and accessible via the submission site. Please log into your account, locate the manuscript record, and check for the action link "View Attachments". If this link does not appear, there are no attachment files.]

To ensure your figures meet our technical requirements, please review our figure guidelines: https://journals.plos.org/plosone/s/figures

You may also use PLOS’s free figure tool, NAAS, to help you prepare publication quality figures: https://journals.plos.org/plosone/s/figures#loc-tools-for-figure-preparation.

NAAS will assess whether your figures meet our technical requirements by comparing each figure against our figure specifications.

# Author comment: Author response to Decision Letter 1

**DOI:** 10.1371/journal.pone.0351326.r002

13 Feb 2026

Dear Dr Miguel Inácio,

We thank you for the opportunity to revise our manuscript and appreciate the constructive feedback from the reviewers, which helped strengthen our methodology and the clarity of the manuscript.

We have addressed all comments raised by the Academic Editor and the reviewers. Below is a point-by-point response detailing how each issue has been resolved.

Response to Academic Editor / Journal Requirements

1. PLOS ONE Style Requirements

Please ensure that your manuscript meets PLOS ONE's style requirements, including those for file naming.

Response: We have carefully reviewed the PLOS ONE style templates and formatted the manuscript, title page, and file names accordingly.

2.Funding Statement and Financial Disclosure

Please provide an amended statement that declares all the funding... Please state what role the funders took... Please remove any funding-related text from the manuscript's Acknowledgements.

Response:

• Thanks for noting the repetition of the information. We have now removed the funding information from the Acknowledgements section of the manuscript.

• The updated and corrected funding statement is the following: “L.Q-U., N.F., H.P., and R.D-C. gratefully acknowledge the support of the German Centre for Integrative Biodiversity Research (iDiv) Halle-Jena-Leipzig, funded by the German Research Foundation (DFG) (DFG–FZT 118, 202548816; https://www.idiv.de/). L.Q-U. was supported by the European Union’s Horizon 2020 research and innovation programme under the Marie Skłodowska-Curie grant agreement No 813904 (https://marie-sklodowska-curie-actions.ec.europa.eu/), and by the project REWILD_DE-2 (funding code 16LW0064K) within the Research Initiative for the Conservation of Biodiversity (FEdA), funded by the German Federal Ministry of Education and Research (BMBF) (https://www.feda.bio/en/). J-S received no specific funding for this work. The funders had no role in study design, data collection and analysis, decision to publish, or preparation of the manuscript. There was no additional external funding received for this study.”

3. Title Page

Please ensure that you include a title page within your main document.

Response: A title page including all authors, affiliations, and the corresponding author has been added to the main document.

6. Supporting Information Captions

Please include captions for your Supporting Information files at the end of your manuscript.

Response: We have added a list of captions for all Supporting Information files at the end of the manuscript.

7. Copyrighted Map Figures (Figures 1, 3, & 4)

We note that Figures 1, 3 & 4 in your submission contain [map/satellite] images which may be copyrighted... We require you to either (1) present written permission... or (2) remove the figures.

Response: We have now carefully checked the licensing of the map layers used in Figures 1, 4, and 5 (formerly Figures 1, 3, and 4). The layers included in these figures and their copyright permissions are as follows:

1. Copernicus (Land Use Data): Fully compatible with CC BY 4.0.

2. Eurostat / GISCO (Administrative Boundaries): CC BY 4.0.

3. Protected Areas: We replaced the previous layers from LUNG M-V (which were only available under CC BY-SA) with the European inventory of Nationally Designated Areas, licensed under CC BY 4.0 (https://creativecommons.org/licenses/by/4.0/). The copyright holder is the European Environment Agency (EEA).

All map layers used in these figures are now fully compatible with the CC BY 4.0 license required by the journal.

Reviewer Comments:

Reviewer #1

1. Figure S1 illustrates very well the steps applied in the study, therefore I would suggest including it in the main part of the text. Response: We agree with this suggestion. We have moved Figure S1 into the main text; it is now labelled as Figure 2

2. Figure S3 is referred in the text as “S2 Figure”. Response: Thank you for spotting this error. We have corrected the citation in the text to refer to the correct figure.

3. Caption of Figure 2: Perhaps the sentence “The circles represent how often specific actions were mentioned in different scenarios” could be removed since the next sentence repeats the same information. Response: We have removed the redundant sentence from the figure caption. It now states as follows: “ Figure 2. Frequency of main actions mentioned by the stakeholders for the different NFF scenarios. Circle size and colour indicate the frequency with which specific actions were mentioned across scenarios: smaller red circles correspond to less frequently mentioned actions, while larger, darker blue circles represent actions cited more often.”

4. Line 386: “The distribution of the three scenarios covered only 1.91%” – does this refer to the overlap between the three scenarios? Response: Yes, this refers to the overlap. We have rephrased this sentence to make it clearer as follows: “ The regions where all three scenarios spatially overlapped on the map covered only 1.91% of the total study area. These areas were found adjacent to natural parks, extensive livestock grasslands, and urban settlements.”

5. Lines 536-537: “The research method demonstrated in this study aided in identifying” - it looks like this sentence is incomplete. Response: Thanks for noting this issue. We have completed the sentence and also improved the readability of the text. This new information can be found on lines 817-826.

6. Discussion: ...Perhaps the authors could elaborate on whether there is any evidence about the acceptance of rewilding among the public and farmers and what actions would be necessary to promote the implementation... Response: We have expanded the Discussion section to address both public and farmer acceptance. The new information can be found in lines 851–862. Specifically, we refer to previous studies from the region that examine acceptance of rewilding and willingness to pay, as well as research highlighting the importance of public education in improving acceptance before implementing rewilding actions. Additionally, we include our reflections on interactions with the rewilding Oder Delta team during the presentation and discussion of outcomes from the participatory process.

Reviewer #2

1. My main criticism concerning the manuscript is that the methodology remains rather abstract... I found the manuscript to be very difficult to follow and understand. Response: We thank the reviewer for highlighting this important issue. We appreciate this feedback and, as a result, have substantially revised the Methodology section to provide greater detail and transparency. The revised text now elaborates on the sequence and structure of the participatory processes, providing clearer descriptions of each methodological step. We have included an expanded explanation of the Net-Map tool, its protocol, and how it was implemented in our study (see lines 200–217). This new content clarifies the rationale for selecting Net-Map, details the steps undertaken during its application, and outlines how the resulting network data were analysed and integrated into subsequent phases of the research. We have also clarified the distinct roles of the stakeholder groups involved in scenario planning and assessment (see lines 177-184), and have incorporated this updated information into Figure 2. These changes make the methodology section clearer and easier to follow, with a full account of our tools and procedures.

2. The local context and need for rewilding the German Oder Delta remain unclear. Furthermore, it should be explained why changes over 30 years are considered in the study area. Response: We have added a new paragraph to the Introduction (lines 114–123) and expanded the Study Area description (lines 142–170) to explain the region’s ecological importance and the specific need for rewilding initiatives. Additionally, we now provide a clear justification for considering a 30-year timeframe, which is consistent with both the Kunming-Montreal Global Biodiversity Framework and the EU Restoration Law (see lines 243–246).

3. There is no information about the applied tool or how the resulting map was used in the study... why it is not presented in the results. Response: We have added a detailed description of the Net-Map tool in the Methodology section and information on how and for what we used the mapping results.

4. A “Net-Map tool” was applied identify stakeholder groups. The resulting network map is presented in supplements. Yet, there is not information about the applied tool or how the resulting map was used in the study. Its is also not clear, why it is not presented in the results. Furthermore, the involvement of stakeholders in the development of the scenarios storylines is mentioned in the methodology (phase 2) and several times in the discussion (Oder Delta experts are not stakeholders use better wording but emphasize it was a collaborative effort to understand local dynamics and future goals to carry out rewilding in the region). Yet, it is not clear who these stakeholders are and if there is an overlap with the interviewed stakeholders. Were the interviewees involved in developing the scenarios? (NO) Or were the scenarios already completed when having the interviews? Response: Thank you for highlighting this important point. We have now added two paragraphs that explain in detail how the Net-Map tool methods were conducted, including the phases involved in their execution (see lines 192–217). We now include the outcomes of the Net-Map tool in both the Results and Discussion sections and describe how this information informed the next steps of the research. We wish to clarify that, in the first iteration of the manuscript, we decided to include information on the Net-Map tool in the supplementary material, as it is not a main outcome of the research but rather a methodological step to facilitate the scenario assessment. We understand that this could be confusing to readers; therefore, we have expanded the text to further explain the processes and how we used the results. However, we have chosen to keep the full network map in the supplementary material, as the key results and their implications are now explained in the Results section, and readers seeking more detailed information can refer to the supplementary files. We believe this approach is appropriate, as the Net-Map is a crucial step in presenting our framework for participatory scenario development, but it is not the main outcome or narrative of the article. Additionally, we provided further clarification regarding the stakeholder groups. Specifically, we clarify that the stakeholders interviewed for the Net-Map analysis were distinct from those selected to assess the already defined scenario storylines. There was no overlap between the two groups. The first group of stakeholders collaborated with us to develop the initial storylines, while the second group participated in assessing and evaluating these storylines through participatory processes. These clarifications improve transparency regarding stakeholder involvement (Lines 177-184 and Figure 2).

5. It is a little confusing that the authors mention four steps in the method, but then refer to a Figure that shows 5 steps. Response: Thanks for noticing this inconsistency. We have aligned the text and the figure. The text now accurately describes the 5 steps shown in the figure (see lines 182-184)

6. Please clarify the terms Narratives, Scenarios, scenario storylines, Actions and Key themes? Response:Thank you for the suggestion. We have added a section in the methods of “Terminology and Definitionsto” clarify the vocabulary used throughout the manuscript ( see lines 177-193)

7. Please review and correct the scales that are shown in the figures. Response: We checked all scale bars from the map figures and they are correct.

8. “S2 Figure” (referred to in Line 159) is missing from the supplementary material. Response:Thank you for noticing this issue. Figure S2 was mistakenly labelled as S3; we have now corrected this error.

9. Lines 237-238: maps seem to be lacking from the S5 Table.Response: We added the map templates used during the interviews; they can be found in Supplementary material SX.

10. Word missing in the caption of Fig. 4.Response: We have corrected the caption for Figure 4.The new captions state as follows: “Consolidated map illustrating the spatial distribution of areas designated for biodiversity conservation, rewetting, blue-green corridors, and biodiversity-friendly agriculture across various scenario narratives. The colours represent each Nature Future Scenario: green for “Nature for Nature,” blue for “Nature for Society,” and yellow for “Nature as Culture.” Darker shades indicate areas identified by 8 or 9 stakeholders, while lighter shades represent areas identified by 1 or 2 stakeholders. B. Map illustrating the intersection of the three scenario maps found in section A. Areas are categorised based on their degree of spatial or physical overlap among the three scenarios, showing distinct zones associated with only one scenario, areas of intersection between two scenarios, and regions where all three scenarios converge.”

11. Meaning of different colours 3 and 4A should be explained. Response: We have added a legend/explanation for the colours in the caption of Figures 3 and 4A. (see lines 499 - 502 and 508-510)

12. Be consistent in the use of American or British English.Response: Thanks for noticing this issue. We have standardised the manuscript to British English.

13. L398: correct “stakeholders” / L403 – correct “on a large scale studies”.Response: Thanks for noticing these errors; they have now been corrected.

Attachment Submitted filename: response reviews PONE-D-25-51781.docx

# Peer review: Decision Letter 1

**DOI:** 10.1371/journal.pone.0351326.r003

**Authors:** Miguel Inácio, Miguel Inácio

30 Mar 2026

PONE-D-25-51781R1

Finding Space for Rewilding: Nature Futures Scenarios Reveal Ecological Opportunities Based on Plural Values of Nature from Participatory Processes

PLOS One

Dear Dr. Quintero Uribe,

Thank you for submitting your manuscript to PLOS ONE. After careful consideration, we feel that it has merit but does not fully meet PLOS ONE’s publication criteria as it currently stands. Therefore, we invite you to submit a revised version of the manuscript that addresses the points raised during the review process.

==============================

**ACADEMIC EDITOR:**

**Dear authors, thank you for accommodating all the queries and making all the necessary changes.**

**Please follow the recommendations of Reviewer 2, who has suggested only minor revisions.**

==============================

Please submit your revised manuscript by May 14 2026 11:59PM. If you will need more time than this to complete your revisions, please reply to this message or contact the journal office at plosone@plos.org. When you're ready to submit your revision, log on to https://www.editorialmanager.com/pone/ and select the 'Submissions Needing Revision' folder to locate your manuscript file.

Please include the following items when submitting your revised manuscript:

- A letter that responds to each point raised by the academic editor and reviewer(s). You should upload this letter as a separate file labeled 'Response to Reviewers'.
- A marked-up copy of your manuscript that highlights changes made to the original version. You should upload this as a separate file labeled 'Revised Manuscript with Track Changes'.
- An unmarked version of your revised paper without tracked changes. You should upload this as a separate file labeled 'Manuscript'.

If you would like to make changes to your financial disclosure, please include your updated statement in your cover letter. Guidelines for resubmitting your figure files are available below the reviewer comments at the end of this letter.

If applicable, we recommend that you deposit your laboratory protocols in protocols.io to enhance the reproducibility of your results. Protocols.io assigns your protocol its own identifier (DOI) so that it can be cited independently in the future. For instructions see: https://journals.plos.org/plosone/s/submission-guidelines#loc-laboratory-protocols. Additionally, PLOS ONE offers an option for publishing peer-reviewed Lab Protocol articles, which describe protocols hosted on protocols.io. Read more information on sharing protocols at https://plos.org/protocols?utm_medium=editorial-email&utm_source=authorletters&utm_campaign=protocols.

As the corresponding author, your ORCID iD is verified in the submission system and will appear in the published article. PLOS supports the use of ORCID, and we encourage all coauthors to register for an ORCID iD and use it as well. Please encourage your coauthors to verify their ORCID iD within the submission system before final acceptance, as unverified ORCID iDs will not appear in the published article. *Only*  the individual author can complete the verification step; PLOS staff *cannot*  verify ORCID iDs on behalf of authors.

We look forward to receiving your revised manuscript.

Kind regards,

Miguel Inácio

Academic Editor

PLOS One

Journal Requirements:

If the reviewer comments include a recommendation to cite specific previously published works, please review and evaluate these publications to determine whether they are relevant and should be cited. There is no requirement to cite these works unless the editor has indicated otherwise.

Please review your reference list to ensure that it is complete and correct. If you have cited papers that have been retracted, please include the rationale for doing so in the manuscript text, or remove these references and replace them with relevant current references. Any changes to the reference list should be mentioned in the rebuttal letter that accompanies your revised manuscript. If you need to cite a retracted article, indicate the article’s retracted status in the References list and also include a citation and full reference for the retraction notice.

[Note: HTML markup is below. Please do not edit.]

Reviewers' comments:

Reviewer's Responses to Questions

**Comments to the Author**

1. If the authors have adequately addressed your comments raised in a previous round of review and you feel that this manuscript is now acceptable for publication, you may indicate that here to bypass the “Comments to the Author” section, enter your conflict of interest statement in the “Confidential to Editor” section, and submit your "Accept" recommendation.

Reviewer #1: All comments have been addressed

Reviewer #2: All comments have been addressed

**********

2. Is the manuscript technically sound, and do the data support the conclusions?

The manuscript must describe a technically sound piece of scientific research with data that supports the conclusions. Experiments must have been conducted rigorously, with appropriate controls, replication, and sample sizes. The conclusions must be drawn appropriately based on the data presented.

Reviewer #1: Yes

Reviewer #2: Yes

**********

3. Has the statistical analysis been performed appropriately and rigorously?

Reviewer #1: N/A

Reviewer #2: N/A

**********

4. Have the authors made all data underlying the findings in their manuscript fully available?

The PLOS Data policy requires authors to make all data underlying the findings described in their manuscript fully available without restriction, with rare exception (please refer to the Data Availability Statement in the manuscript PDF file). The data should be provided as part of the manuscript or its supporting information, or deposited to a public repository. For example, in addition to summary statistics, the data points behind means, medians and variance measures should be available. If there are restrictions on publicly sharing data—e.g. participant privacy or use of data from a third party—those must be specified.

Reviewer #1: Yes

Reviewer #2: Yes

**********

5. Is the manuscript presented in an intelligible fashion and written in standard English?

PLOS ONE does not copyedit accepted manuscripts, so the language in submitted articles must be clear, correct, and unambiguous. Any typographical or grammatical errors should be corrected at revision, so please note any specific errors here.

Reviewer #1: (No Response)

Reviewer #2: Yes

**********

6. Review Comments to the Author

Please use the space provided to explain your answers to the questions above. You may also include additional comments for the author, including concerns about dual publication, research ethics, or publication ethics. (Please upload your review as an attachment if it exceeds 20,000 characters)

Reviewer #1: I acknowledge that the manuscript has been significantly improved, and I would like to thank the authors for carefully considering the reviewers’ comments. However, before publication, I would recommend that the authors carefully review the text to avoid repetitions in both phrasing and content, particularly in the introduction.

Reviewer #2: The authors have improved the comprehensibility of the methodology and provided more clarity by defining used terms.

There are still a few points that require minor revisions:

Line 200 should be: "...which consisted of two"

Line 379: correct "Deltaand"

Line 380: should it be strength or strengths?

In Table 2: Please explain the meaning of two numbers (e.g., 12;3 and 8;1) indicated for Netmap Relevance and Relevance for some interviewees.

**********

7. PLOS authors have the option to publish the peer review history of their article (what does this mean?). If published, this will include your full peer review and any attached files.

If you choose “no”, your identity will remain anonymous but your review may still be made public.

**Do you want your identity to be public for this peer review?**  For information about this choice, including consent withdrawal, please see our Privacy Policy.

Reviewer #1: No

Reviewer #2: No

**********

[NOTE: If reviewer comments were submitted as an attachment file, they will be attached to this email and accessible via the submission site. Please log into your account, locate the manuscript record, and check for the action link "View Attachments". If this link does not appear, there are no attachment files.]

To ensure your figures meet our technical requirements, please review our figure guidelines: https://journals.plos.org/plosone/s/figures

You may also use PLOS’s free figure tool, NAAS, to help you prepare publication quality figures: https://journals.plos.org/plosone/s/figures#loc-tools-for-figure-preparation.

NAAS will assess whether your figures meet our technical requirements by comparing each figure against our figure specifications.

# Author comment: Author response to Decision Letter 2

**DOI:** 10.1371/journal.pone.0351326.r004

1 May 2026

Comments Reviewer #1

1. "I acknowledge that the manuscript has been significantly improved, and I would like to thank the authors for carefully considering the reviewers’ comments. However, before publication, I would recommend that the authors carefully review the text to avoid repetitions in both phrasing and content, particularly in the introduction. Response: Thank you for highlighting this important aspect. We have now thoroughly reviewed the manuscript, with particular attention to the introduction. We removed redundant phrasing and repeated concepts, streamlined the flow of ideas to enhance clarity and conciseness, and ensured that each concept is introduced and developed only once, avoiding unnecessary repetition. These changes are mainly reflected in L42-46; L68-71; L73-95; L110-122.

Comments to Reviewer #2

1. Line 200: Grammar correction "...which consisted of two". Response: The text has been corrected as suggested.

2. Line 379: Typo ("Deltaand"). Response: Fixed to "Delta and".

3. Line 380: "Strength" vs. "strengths". Response: Revised to "strengths" for grammatical accuracy.

4. Table 2: Clarification of notation (e.g., 12;3 and 8;1). Response: We have added a footnote to Table 2 to explain the format as follows: “* two numbers indicate two different actors, each with their own values for mapped relationships and relevance”.

Attachment Submitted filename: response_reviews_PONE-D-25-51781_auresp_2.docx

# Editor report: Decision Letter 2

**DOI:** 10.1371/journal.pone.0351326.r005

**Authors:** Miguel Inácio, Miguel Inácio, Miguel Inácio

26 May 2026

Finding Space for Rewilding: Nature Futures Scenarios Reveal Ecological Opportunities Based on Plural Values of Nature from Participatory Processes

PONE-D-25-51781R2

Dear Dr. Quintero Uribe,

We’re pleased to inform you that your manuscript has been judged scientifically suitable for publication and will be formally accepted for publication once it meets all outstanding technical requirements.

Within one week, you’ll receive an e-mail detailing the required amendments. When these have been addressed, you’ll receive a formal acceptance letter and your manuscript will be scheduled for publication.

An invoice will be generated when your article is formally accepted. Please note, if your institution has a publishing partnership with PLOS and your article meets the relevant criteria, all or part of your publication costs will be covered. Please make sure your user information is up-to-date by logging into Editorial Manager at Editorial Manager® and clicking the ‘Update My Information' link at the top of the page. For questions related to billing, please contact billing support.

If your institution or institutions have a press office, please notify them about your upcoming paper to help maximize its impact. If they’ll be preparing press materials, please inform our press team as soon as possible -- no later than 48 hours after receiving the formal acceptance. Your manuscript will remain under strict press embargo until 2 pm Eastern Time on the date of publication. For more information, please contact onepress@plos.org.

Kind regards,

Miguel Inácio

Academic Editor

PLOS One

Additional Editor Comments (optional):

Dear authors,

Thank you for addressing all aspects from the reviewers.

I consider now the manuscript ready to be accepted.

I would like to call the attention however, in the introduction pleas write the acronyms first time in extent, e.g., EU, IPBES, GBF.

Please correct this in the proofing stage.

Reviewers' comments:

# Editor report: Acceptance letter

**DOI:** 10.1371/journal.pone.0351326.r006

**Authors:** Miguel Inácio, Miguel Inácio, Miguel Inácio

PONE-D-25-51781R2

PLOS One

Dear Dr. Quintero-Uribe,

I'm pleased to inform you that your manuscript has been deemed suitable for publication in PLOS One. Congratulations! Your manuscript is now being handed over to our production team.

At this stage, our production department will prepare your paper for publication. This includes ensuring the following:

* All references, tables, and figures are properly cited

* All relevant supporting information is included in the manuscript submission,

* There are no issues that prevent the paper from being properly typeset

You will receive further instructions from the production team, including instructions on how to review your proof when it is ready. Please keep in mind that we are working through a large volume of accepted articles, so please give us a few days to review your paper and let you know the next and final steps.

Lastly, if your institution or institutions have a press office, please let them know about your upcoming paper now to help maximize its impact. If they'll be preparing press materials, please inform our press team within the next 48 hours. Your manuscript will remain under strict press embargo until 2 pm Eastern Time on the date of publication. For more information, please contact onepress@plos.org.

You will receive an invoice from PLOS for your publication fee after your manuscript has reached the completed accept phase. If you receive an email requesting payment before acceptance or for any other service, this may be a phishing scheme. Learn how to identify phishing emails and protect your accounts at https://explore.plos.org/phishing.

If we can help with anything else, please email us at customercare@plos.org.

Thank you for submitting your work to PLOS ONE and supporting open access.

Kind regards,

PLOS ONE Editorial Office Staff

on behalf of

Dr. Miguel Inácio

Academic Editor

PLOS One
